# Managing disrupted supply chains in Swedish hospitals during the COVID-19 pandemic

**DOI:** 10.1080/20476965.2024.2349816

**Published:** 2024-05-07

**Authors:** Ritva Rosenbäck, Björn Lantz, Peter Rosén

**Affiliations:** a Department of Engineering Science, University West, Trollhättan, Sweden; b Technology Management and Economics, Chalmers University of Technology, Gothenburg, Sweden; c Department of Business Administration, University of Gothenburg, Gothenburg, Sweden

**Keywords:** Supply chain disruption, healthcare, COVID-19, supplies, inventory

## Abstract

The COVID-19 pandemic has disrupted global supply chains and caused widespread shortages of healthcare supplies. This mixed-methods study examined the management of supply shortages in Swedish hospitals during the COVID-19 pandemic. The key findings are that the shortage of supplies was initially severe owing to low regional and national contingency inventory levels, a lack of knowledge of supply chain management, and cost-saving measures. The perceived consequences of the shortage of personal protective equipment persisted in emergency and inpatient departments, in the later waves. Although pharmaceutical shortages later decreased, hospital managers were disappointed that they persisted. This study also highlights the effectiveness of consensus-based hourly supply agreements between service organisations and unit managers, which makes the prioritisation of the limited supply more dynamic. Despite these challenges, hospitals were able to adapt to the supply chain disruptions caused by the pandemic; however, the results emphasise the importance of preparedness for future pandemics.

## 1. Introduction

Supply chain management (SCM) is an important aspect of healthcare delivery (Ziat et al., 2022). During abnormal conditions, such as pandemics, the demand for supplies increases sharply and supply chains become disrupted. Pandemic outbreaks typically have a substantial impact on overall healthcare delivery (Rodriguez et al., 2022). On Invalid Date NaN , the World Health Organization (WHO) warned of a severe and growing disruption in the global supply of personal protective equipment (PPE), which would limit access to supplies such as gloves, medical masks, goggles, face shields, gowns, and aprons. At that point, the WHO estimated a global monthly consumption of 89 million masks, 76 million gloves, and 1.6 million goggles (WHO, 2020). When the Wuhan region, where most worldwide PPE is produced, was locked down at the beginning of the pandemic, production stopped for over two months (Beaulieu et al., 2020), which simultaneously terminated all mask exports (Friday et al., 2021). Therefore, deliveries of masks and other supplies became low. For example, only 6% of the ordered surgical masks and less than 1% of the ordered N95 masks and pairs of gloves were delivered to Canada’s healthcare system early in the pandemic (Zimonjic, 2020). Furthermore, transportation was lacking or delayed, and customs clearance, particularly in China, was severely delayed (Jarrett et al., 2022).

Pandemics, which are rare and have a higher global impact than epidemics and natural and manmade disasters, require special supply chain adaptations (Friday et al., 2021). However, the strategies used to manage supply shortages during COVID-19 were mostly implemented before the pandemic and were designed to deal with disruptions under normal conditions or short-duration crises. Hence, it is necessary to explore preventive strategies for coping with disruptions under abnormal conditions (Ahlqvist et al., 2023; Bhaskar et al., 2020; Jarrett et al., 2022). Ahlqvist et al. (2023) also highlighted a lack of understanding regarding why some nations were able to avoid supply shortages with limited regulatory modifications during the pandemic, whereas others were forced to make drastic adaptations to achieve similar results. This emphasises the need for country-specific research to better understand these phenomena (Beaulieu et al., 2022). Moreover, numerous ongoing studies worldwide have focused on strategic supply chain management in healthcare during the COVID-19 pandemic, where various supply chain settings have been explored to manage the increased demand for essential supplies on a strategic level. However, few papers discuss how supply shortages are managed on an operational level and no papers were found that have studied how first- and second-line hospital managers perceived the management of the supply shortages. Understanding such perceptions is crucial because such managers are at the forefront of implementing and adapting supply chain strategies in real-time, under the pressures of a global health crisis. The insights of these managers are instrumental for assessing and improving supply chain strategies under crisis conditions. Their experiences offer practical insights for developing resilient supply chain frameworks, vital for future disruptions. Therefore, this study explores how hospital managers in Sweden perceived the management of supply shortages during the COVID-19 pandemic.

The remainder of this paper is organised as follows: The next section provides a literature review of supply chain management in healthcare during the COVID-19 pandemic. Thereafter, the research methodology is presented, followed by a presentation of the results. Finally, the results are discussed, and conclusions and recommendations for future research are presented.

## 2. Literature review

Many studies have reported supply shortages in hospitals; for example, PPE, consumables, technical equipment, and pharmaceuticals were at dangerously low inventory levels at the beginning of the pandemic (Bhaskar et al., 2020; Capuzzo et al., 2022; Friday et al., 2021; Jarrett et al., 2022; Raunig et al., 2020; WHO, 2020). The shortage of PPE resulted in rising prices (Jarrett et al., 2022); for example, the price of surgical masks increased six-fold in April 2020 (Shopp.org, 2020; WHO, 2020). Moreover, COVID-19 patients’ need for intensive care caused a shortage of technical equipment, including ventilators, respirators, and dialysis equipment (Jarrett et al., 2022). The demand for ventilators in 2019 rose sharply at the beginning of 2020 (Bhaskar et al., 2020; Center for Health Security, 2022), with the number of installations at one hospital in northern Italy increasing more than five times between 2019 and May 2020 (Capuzzo et al., 2022). In addition, the increased need for intensive care suddenly increased the demand for the required pharmaceuticals, and the shortage became severe (Jarrett et al., 2022).

There are several reasons for these supply shortages, such as low initial inventory levels, a sudden rise in demand, low supply from suppliers, and a lack of trust between supply chain stakeholders (Bhaskar et al., 2020). Beaulieu et al. (2022) discussed critical areas of improvement to better prepare for future unforeseen situations, such as inventory management, SCM, and collaboration between supply chain and infection prevention department managers, as well as between healthcare institutions and suppliers. Several authors claim that contingency inventory levels worldwide have decreased over several years owing to cost savings and a relatively calm and open world (Ahlqvist et al., 2023). Moreover, the implementation of lean-based management further reduces inventory levels (Friday et al., 2021; Jarrett et al., 2022) and may be an inappropriate tool for health organisations to improve their financial and operational performance due to the importance of preparing for crises (Bhaskar et al., 2020).

The endeavour to reduce the cost of materials has led to increased single sourcing and the use of third-party logistics providers and foreign suppliers, especially those in China (Ahlqvist et al., 2023; Jarrett et al., 2022; Torsekar, 2020). For example, Canadian PPE supplies are almost entirely dependent on foreign markets (De Montigny, 2020), and Australia imports 90% of its pharmaceutical needs (Coorey, 2020). The delivery of medicines unprotected by patents and raw materials for pharmaceuticals, which are often produced in China, has been severely affected (Bhaskar et al., 2020).

Deficient inventory control systems and the increased use of third-party logistics providers have sometimes led to hospital managers losing control of their inventory levels (Ahlqvist et al., 2023; Beaulieu et al., 2022). Therefore, during the pandemic, some hospitals established inventory control systems to visualise their organisation’s inventory levels (Jarrett et al., 2022). Ash et al. (2022) highlighted the importance of surveillance and early warning systems at the beginning of a pandemic. Sharing risk information regarding inventory disruptions, supply transit, and spillover effects can support the most exposed areas at each significant moment (Friday et al., 2021). Some management organisations have created daily visual management tools, such as traffic light colour codes, to provide signals when supplies reach low levels (Jarrett et al., 2022).

Several scholars have discussed various models for increasing supply chain reliability, including predictive big data analysis, queuing theories, and blockchain connectors (Bhaskar et al., 2020; Friday et al., 2021). However, at the beginning of the pandemic, when purchasers panicked, governments closed their borders, and transportation systems broke down, it became difficult to find accurate information on inventory levels, orders, delivery times, and delivery possibilities, which were needed for the use of inventory control systems (Beaulieu et al., 2022; Friday et al., 2021). Understanding the effects of the pandemic requires enhancing inventory management knowledge of the uncertainty and risks involved in end-user behaviour and decision-making in situations where uncertainty and risk probabilities cannot be estimated (Friday et al., 2021).

Therefore, there is a need for SCM expertise in healthcare (Bhaskar et al., 2020). Ash et al. (2022) emphasise the potential of a resilient approach to supply chain management in an environment of disruptions rather than concentrating on short-term efficiencies. Examples of measures to improve long-term efficiency include multiple sources, alternative transportation routes, and increased safety stock levels (Ahlqvist et al., 2023). SCM experts should also be allowed to trigger contingency plans such as locking contracts, reinforcing logistical capacities, and drawing from emergency stock (Ash et al., 2022). However, most healthcare organisations have limited knowledge of logistics and inventory strategies. In addition, when the risk of shortage increased, purchasing was partly centralised to governments, which placed the orders, while regions and hospitals made purchases according to their usual routines, thereby overcrowding the suppliers’ order processes (Friday et al., 2021).

According to Beaulieu et al. (2022), there is also a need for national supply chain command and distribution centres. Jarrett et al. (2022) claimed that smaller hospitals and communities are more dependent on collaboration and centralisation during a pandemic. Collaboration between supply managers, infection prevention department managers, and healthcare suppliers is essential to obtain a more accurate view of the situation and develop better solutions during a shortage (Beaulieu et al., 2022). During a pandemic, SCM requires trust, commitment, power, and information sharing to achieve a satisfactory performance (Friday et al., 2021).

Many articles have discussed the approaches adopted in different supply chain settings to manage the surge in demand for materials, but mostly at a strategic level, therefore our research focuses on the operational level. However, some papers describe local initiatives, such as those by well-established manufacturers (e.g., Tesla, General Motors, Ford, Dyson, and Rolls-Royce), who have repurposed their existing facilities to meet the rising demand for non-ventilator products (Bhaskar et al., 2020). In Sweden, Absolut Vodka started production of hand disinfectant (“Vodkajätten ställer om till handsprit”, 2020), while several other companies produced PPE using 3D printers (Jarrett et al., 2022). Hospitals also established committees to prioritise the use of a limited amount of test equipment between stakeholders, and healthcare providers held daily meetings with supply chain staff to communicate changing policies and recommendations during the initial stages of the pandemic (Jarrett et al., 2022). Instructions on how to make masks and hand sanitisers appeared online, and several healthcare practitioners began to reuse PPE when they were unsure when the next delivery would be (Bhaskar et al., 2020).

## 3. Materials and methods

### 3.1. Design

This study used a mixed-methods approach to gain a comprehensive understanding of the supply situation in Swedish hospitals during the COVID-19 pandemic. Specifically, a preliminary qualitative case study was used to provide information for the principal quantitative survey, as per Morgan’s (1998) priority-sequence model. This design was adopted due to the lack of suitable previous literature that could be used to develop a questionnaire with the specific orientation required for this study (see AlRuthia et al., 2023; Vann Yaroson et al., 2023 for similar approaches). An inductive approach was employed to analyse data from 39 interviews in two case studies regarding shortages of consumables and equipment during various waves of the pandemic. The results of the qualitative study offer an overview of the supply situation and its management, which were subsequently generalised in a web-based questionnaire administered to managers in intensive care units (ICUs), emergency departments (EDs), and inpatient units at hospitals across Sweden. All questionnaire items were derived from the interview results, and the questionnaire was pretested in a pilot study, where it was reviewed by two experts and thereafter by five actual respondents from the target population. Minor improvements were made at each step.

### 3.2. Setting

To generate informative and diverse results, two distinct cases were selected for the qualitative study (Eisenhardt, 1989). Case A involved a medium-sized hospital with approximately 1300 employees located in a region with high population density. The region had several more hospitals and experienced an early and intense COVID-19 outbreak. The hospital had no infection facilities and only a few intensive care beds. The number of intensive care beds increased 3.5 times during the beginning of the pandemic. In contrast, Case B featured a larger (approximately 5000 employees) central hospital in a region with a lower population density and only two more hospitals.COVID-19 arrived later in this region and had a less severe impact. This hospital had a regional infection department and a sufficient number of intensive care beds. Both regions had more than two hospitals that collaborated with each other during the pandemic, and patients were transferred between these facilities. Both cases used third-party logistics providers for consumable and pharmaceutical inventory management under normal conditions and did not have in-house inventory or supply managers.

### 3.3. Data collection

To attain a representative sample in the descriptive study, semi-structured interviews were conducted with all managers designated by the hospital administration to be responsible for the COVID-19 units. In Case A, 25 individuals were interviewed, including representatives from the hospital management team, the Chief Executive Officer (CEO) and Chief Medical Officer (CMO), and managers from the operations, communication, human resources, education, care, ICU, ED, inpatient care, logistics, and maintenance departments. Case B, wherein some departments were operating as regional departments, had a different selection of managers. Eleven interviews were conducted with the regional director of health and medical care, the manager of the regional crisis management staff, the chief of staff, the chief physician and chief hygiene physician, and managers from the ED, infection, internal medicine, and service departments. All interviewees provided written consent.

Semi-structured interviews were conducted episodically to encourage respondents to speak more freely (Gioia et al., 2013), but an interview guide was utilised to support their memory and prevent any important information from being omitted. The interview guide was developed from the literature and initial interviews with managers. During the interviews, questions about the longitudinal change, for example, differences between the first and later waves, was covered. All interviews were recorded, transcribed, and analysed.

### 3.4. Questionnaire development

Drawing from the results of the qualitative study, the quantitative study aimed to i) closely investigate the extent of the material shortage problem in Sweden, ii) validate the findings of the qualitative study, iii) investigate whether the shortages were resolved between the first and later waves, and iv) examine the differences between ICU managers and other unit managers like ED and inpatient care.Earlier research has described a much higher pressure in the ICU than in other units. The impact of a found lower-than-normal emergency demand and lowering the elective care saved other units from pressure but did not affect the ICU to the same extent (Rosenbäck & Svensson, 2023). Therefore, there was interest in separating ICU from other units in the questionnaire. The questionnaire included introductory questions, such as the region in which the respondent worked, and their department and management role, and comprised nine sections and 58 items. This study focused on two of these sections, which contained questions about PPE, consumables, equipment, and pharmaceuticals (the other sections covered areas such as staffing, contingency plans, meeting routines, digitalisation, information flow, and training).Each item in these two sections, excluding the introductory questions, appeared in two versions; one for the first and one for later waves of the pandemic, with the purpose to identify changes or trends in the situation over time. Table 1 lists the items that were used to compare the first and later waves. The items for the first wave are numbered from 1 to 6, whereas those for the later waves are numbered from 7 to 12. Numbers 13 (first wave) and 14 (later waves) were the used dependent items. According to the qualitative study, supply problems included PPE, consumables, technical equipment, and pharmaceuticals. Items 1, 4, 5, and 6 concern the shortage levels for different types of materials. The questionnaire included six dependent items, of which one was used here (in the same two versions as mentioned above), namely, the respondents’ level of satisfaction with the management of supplies during the pandemic. Each question was rated on a five-point Likert scale (1 = strongly disagree; 5 = strongly agree) to offer a balanced and straightforward approach to capturing participant responses. This scale design is assumed to reduce the cognitive load on respondents while providing sufficient variance for meaningful statistical analysis (Jenkins & Taber, 1977).

**Table 1.** Questionnaire items.

Item	Statement
1	We had a shortage of protective equipment during the first wave.
2	We used more secure protective equipment than recommended during the first wave.
3	We used less secure protective equipment than recommended during the first wave.
4	We had a shortage of consumables such as sampling swabs, breathing circuits, etc. during the first wave.
5	We had a shortage of technical equipment during the first wave
6	We had a shortage of pharmaceuticals for COVID-19 treatment during the first wave.
7	We had a shortage of protective equipment during later waves.
8	We used more secure protective equipment than recommended during later waves.
9	We used less secure protective equipment than recommended during later waves.
10	We had a shortage of consumables, such as sampling swabs, breathing circuits, etc. during later waves.
11	We had a shortage of technical equipment during later waves.
12	We had a shortage of pharmaceuticals for COVID-19 treatment during later waves.
13	I am satisfied with the way the hospital managed the supplies and equipment during the first wave.
14	I am satisfied with the way the hospital managed the supplies and equipment during later waves.

The target population for the survey was first- and second-line managers of Swedish hospital departments and units responsible for treating patients with COVID-19. These managers were responsible for operations but not the supply of materials. Participation from all 21 regions in Sweden was requested. The researchers shared a link to the questionnaire and information about the study with 773 ICU, ED, and inpatient care unit managers via email.

A multifaceted approach was employed to assess the potential impact of non-response bias on the study’s findings (Wagner & Kemmerling, 2010). First, assuming that late respondents were more likely to be non-respondents, we performed an extrapolation analysis by comparing those who responded after the second reminder (late respondents) with those who responded before the reminder (early respondents) using a Mann – Whitney U-test based on the Likert items. Second, we compared the actual distribution of the types respondents at the population level with the sample distribution to assess consistency. Finally, a subset of non-respondents was contacted to understand the reasons for not participating in the survey, even after multiple reminders were sent.

### 3.5. Data analysis

The qualitative data were subjected to inductive coding using NVIVO12 software to obtain a comprehensive understanding of the data (Eisenhardt et al., 2007) and theoretically describe the phenomena under investigation (Gioia et al., 2013). To achieve this, the researchers collaboratively established first-order codes and then organised them into themes. The themes used in this study were PPE, consumables, equipment, and pharmaceuticals, which were subsequently divided based on shortage severity, shortage management, and concerns of the employees during the pandemic.

The questionnaire data were subjected to exploratory analysis given the inductive nature of the study. A descriptive analysis was initially conducted to distinguish between the various sub-questions. Subsequently, the data were further analysed using principal component analysis (PCA) with varimax rotation. PCA is a statistical technique for reducing a large number of variables in a dataset into a small number of unrelated principal components to increase the interpretability of the data while preserving the maximum amount of information (Field, 2013). Bartlett’s test of sphericity and the Kaiser – Meyer – Olkin (KMO) measure of sampling adequacy were employed to evaluate the suitability of the PCA (Faul et al., 2009).

Multiple regression analyses were performed to examine the relationship between the managers’ satisfaction with the hospital’s management of supplies and equipment, and various categories of supplies and equipment. It should be noted that parametric methods, such as t-tests and regression analysis, are known to be robust in terms of violations of the general assumptions (e.g., normality and homoscedasticity) on which these methods formally rely (Carifio & Perla, 2008; Norman, 2010). The nominal significance level was 0.05; however, the Bonferroni – Holm method (Holm, 1979) was used to control for the family-wise error rate, thereby mitigating the risk of alpha error inflation.

To ascertain the principal factors influencing manager satisfaction, we employed a backward stepwise regression technique to select the relevant variables. This technique eliminates variables that do not contribute significantly, resulting in a more streamlined regression model. During each iteration of the elimination procedure, the independent variable with the highest p-value was removed from the model. This recurring sequence was maintained for as long as there was at least one predictor variable with a p-value of > 0.100, thus arriving at a parsimonious model. This approach aligns with the exploratory nature of this study (Hair et al., 2014).

## 4. Results

### 4.1. Qualitative study

The qualitative study was the preliminary study for constructing the questionnaire and is able to explain some of the quantitative results. A summary of the results from the qualitative study is presented here, and proof excerpts are found in the Supplementary Materials.

The first major problem that the interviewees experienced during the pandemic was severe material shortages. Case A, which experienced an earlier outbreak, initially had more problems than Case B, and the results therefore focus on Case A. Initially, the managers of both Cases A and B described the risk of a PPE shortage as severe, which affected the hospitals’ ability to provide healthcare because the employees were concerned about COVID-19 infection. Additional material shortages impacting consumables, surgical equipment, and pharmaceuticals were also reported in the interviews. When laboratory infection analysis methods became faster and laboratory capacity increased, the demand for sampling consumables also increased, which caused the next severe shortage. The delivery situation seemed to improve later in the pandemic, and demand was nearly fulfilled, especially for PPE and consumables.

The ICU had a higher demand for technical equipment and pharmaceuticals than other departments in both cases. In Case A, the ICU managers described the development of an inventive environment with no economic limits, and they quickly identified alternative working methods, equipment, pharmaceuticals, and PPE. The hospital in Case A was the first to ask the government about using gasmasks from the contingency inventory, which were needed in the ICU; thereafter, gasmasks were delivered to all Swedish hospitals, as in Case B.

During the interviews, discussions emerged regarding whether the staff used safer or less-safe PPE. At the beginning of the pandemic, when knowledge was low and safety recommendations changed frequently, staff demanded safer PPE than was recommended. After some time, when the inventory situation had improved and the infection transmission routes were better known, the direness of the situation decreased. This improved the possibility of making individual choices regarding the PPE safety level, wherein a small proportion of the staff chose to exceed the recommended safety levels. At the end of the third wave, some interviewees mentioned that staff used less safe PPE than recommended because they had already been infected and/or were vaccinated. The ICU was prioritised and used safer PPE (such as gas masks) than other units owing to aerosol-forming tasks. This prioritisation caused some dissatisfaction, and staff outside the ICU felt less valued.

To cope with material shortages in both cases, disposable items for ventilators were cleaned and stored for use in emergency scenarios owing to significantly reduced or completely halted supplies. The managers in Case A attempted to resolve the pharmaceutical shortage by purchasing old-fashioned pharmaceuticals, pharmaceuticals from other countries, and veterinary pharmaceuticals.

In Case A, due to shortages during the first wave, the material orders from all units became extremely high because managers wanted to ensure that their colleagues had sufficient PPE. They also experienced a period of theft and were forced to lock up their storerooms. To determine how to best distribute the materials to those who needed them most, the service organisation and unit managers calculated what each unit needed per hour and began to make deliveries around the clock. They also tried to continuously increase the safety stock until they had reached a stock level that covered a few days’ needs.

### 4.2. Questionnaire results

The response rate for the quantitative study was 35.2% (272/773), which is similar to that of related studies (Baruch & Holtom, 2008). A high response rate can minimise the risk of non-response bias (Groves & Peytcheva, 2008); however, it is still necessary to evaluate such risks. The results of the non-response analysis are as follows. First, the Mann – Whitney U-tests revealed that eight out of 126 tests required to evaluate all Likert items in the questionnaire were significant, which falls within the expected range for type I errors, suggesting that late respondents did not significantly differ from early respondents. Second, the distribution of the types of materials managed by the sample respondents was similar to the same distribution at the population level. Finally, all sampled non-respondents indicated that their non-participation was due to lack of time, not being a relevant manager, or because the questionnaire was marked as spam. No indication of systematic bias was observed. Based on these analyses, we concluded that non-response bias was unlikely to be a concern in this study (Wagner & Kemmerling, 2010).

The descriptive statistics for all 14 questionnaire items are presented in Table 2. The items were arranged in pairs to enable easier comparisons between the first and later waves of the pandemic. Table 2 also lists the p-values from the t-tests to examine the difference in mean values for each pair of items (Items 1 and 7, Items 2 and 8, etc.) separately for the ICU and other units. These tests indicated that after Bonferroni – Holm correction, there were significant differences for all pairs in both the ICU and other units. This was because the situation during the first wave of the pandemic was unfamiliar and more severe than that of the later waves. Overall, the results showed that the situation regarding supplies and equipment was more challenging in the ICU than in other units during the first wave. However, this effect was largely reduced or eliminated during later waves.

**Table 2.** Descriptive statistics and pairwise comparisons (the items are described in Table 1).

	ICU				Other units				ICU vs. other units
Item	Mean	S.D.	*n*	*p*	Mean	S.D.	*n*	*p*	*p*
1	3.68	1.24	85	<0.001	3.34	1.43	181	<0.001	0.056
7	1.57	0.83	86		1.48	0.84	179		0.419
2	2.80	1.51	84	0.003	2.73	1.41	180	<0.001	0.737
8	2.16	1.24	85		2.10	1.21	178		0.693
3	1.26	0.71	84	0.019	1.56	1.14	179	<0.001	0.026
9	1.06	0.29	82		1.13	0.48	178		0.231
4	3.61	1.26	83	<0.001	3.05	1.27	168	<0.001	0.001
10	1.98	0.94	84		1.79	0.93	165		0.146
5	3.12	1.52	84	<0.001	2.42	1.30	170	<0.001	<0.001
11	1.65	0.81	84		1.53	0.80	167		0.235
6	3.35	1.29	80	<0.001	2.05	1.21	144	<0.001	<0.001
12	1.63	0.75	79		1.52	0.83	145		0.303
13	3.08	1.36	85	<0.001	3.39	1.25	179	<0.001	0.069
14	4.12	0.96	84		4.34	0.88	178		0.071

To examine the data further, four PCAs were performed using the varimax rotation method. The PCAs were performed on two sets of items (Items 1–6 and 7–12) and were conducted separately for the ICU and other units. The results of Bartlett’s test of sphericity indicated statistical significance in all four cases (*p* < 0.001); however, the KMO measure of sampling adequacy ranged between 0.613 and 0.756, which may not be ideal for PCA analysis (Field, 2013). Additionally, the factor loadings were low, and the reliability measures (Cronbach’s alpha, see Field, 2013) for the resulting factors were unsatisfactory. Details of these PCAs are provided in the Supplementary Materials. Therefore, an analysis based on a single item was the most appropriate approach.

A multiple linear regression analysis using Items 1–6 as independent variables and Item 13 as the dependent variable was conducted to analyse managers’ satisfaction with the way their hospital managed supplies and equipment in the ICU during the first wave. The final model contains one explanatory variable. As shown in Table 3 (Columns 2–4), the shortage of protective equipment was significantly and negatively associated with managers’ satisfaction with how the hospital managed supplies and equipment during the first wave.

**Table 3.** Regression analyses results regarding managers’ satisfaction with the way their hospital managed supplies and equipment tThe items are described in Table 1).

	First wave						Later waves					
	ICU			Other units			ICU			Other units		
Item	B	S.E.	*p*	B	S.E.	*p*	B	S.E.	*p*	B	S.E.	*p*
(Constant)	4.420	0.459	<0.001	4.542	0.243	<0.001	5.038	0.240	<0.001	5.503	0.204	<.001
1	−0.366	0.118	0.003	−0.191	0.072	0.009						
3				−0.331	0.094	<0.001						
7										−0.240	0.098	0.015
9										−0.263	0.141	0.063
10										−0.282	0.087	0.002
12							−0.582	0.132	<0.001			
F	9.664			15.798			19.360			15.494		
*p*	0.003			<0.001			<0.001			<0.001		
R^2^	0.116			0.191			0.214			0.260		

A similar multiple linear regression analysis was conducted to analyse managers’ satisfaction with the way their hospital managed supplies and equipment in other units during the first wave. In this case, the final model had two explanatory variables, as shown in Columns 5–7. The shortage of protective equipment and use of less secure protective equipment than recommended were both significantly negatively associated with managers’ satisfaction during the first wave.

Next, a multiple linear regression analysis using Items 7–12 as independent variables and Item 14 as the dependent variable was conducted to analyse managers’ satisfaction with the way the hospital managed supplies and equipment in the ICU during later waves. The final model had one explanatory variable (Columns 8–10). The shortage of pharmaceuticals for COVID-19 treatment was significantly and negatively associated with managers’ satisfaction during the later waves.

The final multiple linear regression analysis, using Items 7–12 as independent variables and Item 14 as the dependent variable, analysed managers’ satisfaction with the way the hospital managed supplies and equipment in other units during the later waves. The final model had three explanatory variables (Columns 11–13). The shortage of protective equipment, the use of less secure protective equipment than recommended, and the shortage of consumables such as sampling swabs and breathing circuits were all significantly negatively associated with managers’ satisfaction during the later waves.

Finally, multicollinearity did not seem to be an issue in any regression model, because each model had a low variance inflation factor (VIF). Details of the backward elimination procedures for all four regression models, including the VIF values, can be found in the Supplementary Materials. All four regression models were significant after Bonferroni – Holm correction.

## 5. Discussion

The mixed-methods approach used in this study, created opportunities for our analysis, because it enabled us to evaluate if the findings in our initial qualitative study were valid on the population level (Morgan, 1998). The structure of the questionnaire, focusing on both the first and later waves of the pandemic, provided us with the opportunity to longitudinally analyse how managers in Swedish hospitals perceived the management of supply shortages, which had not been examined in previous research.

As described by researchers who have examined other countries (Ahlqvist et al., 2023; Bhaskar et al., 2020), Sweden has dismissed the need for a contingency inventory of essential healthcare materials in recent decades, and thus has an exceptionally low national inventory level. Moreover, we identified that SCM was often handled by third-party logistics providers and reduced hospitals’ in-house knowledge and control. This was consistent with the findings of other studies (Jarrett et al., 2022; Torsekar, 2020). The supply of materials at Swedish hospitals was perceived to be more challenging during the first wave than during later waves, and the situation in the ICU was more severe than that in other units. Our qualitative research shows that the two analysed hospitals solved their material shortage problems during the later waves, which was not always the case in other hospitals and countries (Friday et al., 2021; Jarrett et al., 2022).

PPE shortages were at Swedish hospitals significantly negatively associated with managers’ degree of satisfaction with the way their hospital managed the situation in the first wave, both in the ICU and other units. The qualitative study revealed that administrative staff and/or assistants, especially at the hospital in Case A, spent a lot of time cleaning disposable PPE for reuse and producing aprons and visors during the first wave, which is consistent with the findings of other scholars (Bhaskar et al., 2020; Jarrett et al., 2022). PPE is easy to produce, and in Sweden, manufacturers of other products began producing it in 2020, as in other countries (Vodkajätten ställer om till handsprit, 2020; Bhaskar 2020). The shortage of PPE was resolved in the ICU during later waves, but it remained one of the biggest problems perceived by the managers of other units. The findings showed that the hospital in Case B, which was in an area that experienced a later infection start date, had fewer problems than that in Case A, where infections began earlier. For more advanced respiratory protective equipment, Case A fast-tracked the evaluation of existing options in the PPE market, which resulted in multiple options for staff. Moreover, they were the first to push the government to provide gas masks from the contingency inventory, which were later available to other hospitals as required.

Consumable shortages during later waves of the pandemic were at Swedish hospitals significantly negatively associated with mangers’ degree of satisfaction with how their hospital managed supplies and equipment in other units. According to the mean values of the respondents’ judgements, the shortage of consumables seemed to be a problem in the first wave; however, it was perhaps concealed by the more severe PPE shortages. This problem was significantly worse in the ICU than in other units at Swedish hospitals and decreased significantly in the later waves. The high mean values persisted throughout all waves of the pandemic, suggesting that different consumables experienced shortages during the first and later waves of the pandemic, which is consistent with the results of other studies (Capuzzo et al., 2022; Jarrett et al., 2022). According to the qualitative study, employee anxiety during the first wave in Case A was not only caused by the lack of PPE, but also by the lack of breathing circuits, which in turn is likely to have been caused by high intensive care requirements at the beginning of the pandemic. Later, when laboratory capacity increased, the shortage of sampling sticks also became more severe in Case A. Although managers perceived the shortage of consumables to be less urgent during the later waves of the pandemic, satisfaction with how these shortages were managed was higher during the first wave. This highlights managers’ exclusive focus on the severe PPE shortages during the first wave.

At the beginning of the COVID-19 pandemic, there was a shortage of technical equipment for high-flow oxygen treatment and respirators (WHO, 2020; Capuzzo et al., 2022; Jarrett et al., 2022). In this study, the shortage of technical equipment was perceived to be significantly worse during the first wave, both in the ICU and other units at Swedish hospitals. During the first wave, it was also perceived to be significantly worse in the ICU than in the other units; however, the difference became insignificant during later waves. This is not surprising, given the ICU’s higher general dependency on technical equipment. However, according to the qualitative study, the hospital in Case A was able to both buy and borrow equipment during the first wave, which contributed to the effective management of the situation. The situation at the hospital in Case B was not as severe, even in the first wave, because the outbreak was slower and appeared later.

The shortage of pharmaceuticals for the treatment of COVID-19 was significantly negatively associated with managers’ degree of satisfaction with the way their hospitals managed supplies and equipment in the ICU, but only during the later waves. When in the ICU, patients require special pharmaceuticals; according to the qualitative study, pharmaceuticals faced the most severe shortage throughout the entire period, which is in line with the findings of other researchers (Friday et al., 2021). The findings of the qualitative study indicate that there was a pronounced sense of emergency at the beginning of the pandemic, resulting in a higher acceptance of exceptional solutions such as using alternative pharmaceuticals or markets. That managers were more negative about these shortages in later waves is likely because they were dissatisfied that the disruption had not yet been resolved. Difficulties in solving this problem were probably caused by supply chain disruptions in the Chinese pharmaceutical industry (Vila-Parrish et al., 2012). In addition, the evolving understanding of a new infection can rapidly change the need for different pharmaceuticals during a pandemic.

The use of less secure PPE than recommended was significantly negatively associated with managers’ degree of satisfaction with the way their hospital managed the supplies and equipment in other units in all waves. First- and second-line managers were dissatisfied when PPE safety recommendations could not be adhered to or exceeded in the workplace. Staff at Swedish hospitals wanted to feel secure, especially at the beginning of the pandemic, when safety recommendations were frequently being updated. The use of safer equipment was significantly reduced during the later waves in both the ICU and other units. The managers in Case A requested not to use PPE at all during the later waves; this is because the increase in knowledge about COVID-19 and the start of the vaccination programme heightened their sense of security.

The ICU managers at Swedish hospitals did not have the same significant negative association with the use of less secure PPE than recommended, probably because the ICU had been provided with the recommended PPE earlier than other units, as seen in Case A. The differences in PPE provision between the ICU and other units resulted in mistrust and dissatisfaction with hospital management, as managers believed that safer equipment should have been provided for all units, and that the staff in other units should have been as valued as the staff in the ICU.

In Case A, the service organisation overcame a period of insufficient inventory, uncertain supplies, and an overabundance of orders by changing to hourly 24/7 deliveries. The service organisation and unit managers agreed on a calculated hourly consumption rate, which made delivery prioritisation more dynamic. The safety stock slowly built up and increased the possibility of compensating for missed deliveries. Moreover, these actions rapidly increased in-house supply chain knowledge and control. This approach to managing deliveries during a crisis is a key finding of this study.

This study had several limitations. First, we adopted a retrospective survey-based approach, which may have been subject to recall bias and limitations in data accuracy. Additionally, varying response rates across regions may introduce sampling bias, impacting the representativeness of the study’s findings. Second, owing to the exploratory nature of the study, a psychometric analysis of the survey instrument was not conducted, which could have affected the validity and reliability of the data collected. Thirdly, our study does not examine the causes of the shortages. For future pandemic preparedness, a combined focus on these causes and managers’ perceptions is important. Furthermore, the results were specific to hospitals in Sweden and may not be directly generalisable to hospitals in other countries. s. Finally, the study’s reliance on self-reported data may introduce a response bias, potentially influencing the accuracy of the reported information.

## 6. Conclusion

Swedish national and regional contingency stock levels were insufficient owing to the prolonged experience of a relatively calm and open world, many years of cost savings, and a lack of SCM knowledge. This resulted in severe shortages of PPE, consumables, technical equipment, and pharmaceuticals during the COVID-19 pandemic. Although neither of the Swedish case hospitals were completely out of stock, the prolonged supply disruptions experienced during the pandemic were difficult to anticipate. Our findings indicate that there is a need for higher contingency inventory levels to manage the first wave of an infection, and also that flexible production facilities should be used at a pre-agreed contingency production capacity.

Among the shortages examined in this study, the PPE shortage was the most severe and overshadowed managers’ experiences of other shortages. However, in later waves, frustration with unresolved pharmaceutical shortages in the ICU and consumable shortages in other units became evident, even though the situation improved since the first wave. The practical implication of these findings is that a holistic approach addressing all types of shortages from the outset is essential.

During the initial stages of the COVID-19 pandemic, staff requested excessive PPE owing to limited knowledge about the infection, changing safety recommendations, and PPE shortages. If the PPE supply had become critical at the onset of the pandemic, staff would have refuse to work, resulting in the collapse of healthcare provision. Our results show that management must be able to deliver PPE that exceeds the staff’s requirements to minimise the risk of staff refusing to work.

This study differs from other studies on SCM during the pandemic in that it examines both its operational and strategic levels. Similarities were found between these levels, including country-specific egocentricity.

One key finding of the qualitative study was that the shift to just-in-time deliveries based on a calculated hourly demand resulted in the efficient use of limited supplies. The daily presence of the service organisation and its collaboration with unit managers increased the possibility of prioritisation, which was more efficient than planning based on inventory shortages.

Future research is required to create a framework for optimising contingency inventory, contingency production capacity, and the mix of suppliers in healthcare organisations during times of abnormal demand. Moreover, researchers should investigate whether the observed supply method based on local hourly consumption can be applied at higher system levels.

## Supplementary Material

Supplemental Material

## Data Availability

The authors confirm that the data supporting the findings of this study are available within the article [and/or] its supplementary materials.
