# Supplementary material for: Managing disrupted supply chains in Swedish hospitals during the COVID-19 pandemic
Source: Health Syst (Basingstoke). 2024 May 7;14(1):58–68. doi: 10.1080/20476965.2024.2349816 (PMC11843631; doi:10.1080/20476965.2024.2349816)
Supplement: Supplemental Material [file THSS_A_2349816_SM1633.zip › Excerpts.docx]

**Excerpts for qualitative study in paper ”Managing Disrupted Supply Chains in Swedish Hospitals During the COVID-19 Pandemic”**

“You could have what visions you want…. How long time shall we manage with the stock we have? But it cost money” (Chief of staff, case A).

“When it was at worst, it was only hours before we were left without personal protective equipment” (Chief physician, case B) .

“Sometimes it was gloves sometimes it was surgery equipment and pharmaceutics. Yes, we actually have had shortage of nearly everything sometimes like everybody else” (Chief of staff, case B) .

“We need to know if we can clean the hoses to the ventilators, because we do not know if we get any deliveries. It was a very serious situation” (Chief of staff, case B) .

“We do not know if we will have propofol so we could put the patients at ICU to sleep.” (Chief of staff, case B).

” And then we had Chinese and Russian pharmaceuticals without Swedish labels it was only on Russian and Chinese”. (Chief nurse at the ICU, case A).

“And another product with shortage was sampling material” (Chief of staff, case B).

“We kept a lot of consumables to medical devices not to stand without if it (authors remark: deliveries) would fail, but after a while the deliveries caught up with the demand” (Manager of the ICU, case A).

“Consumables and material supplies stabilized considerably during summer and autumn (authors remark 2020), but still it was very uncertain, uncertain prognosis” (Chief physician, case B).

“It was hard when we needed to change- it comes new recommendations all the time and you had to say this is what we shall use and then no, now it is this we should use” (Chief nurse of the Emergency department, Case A.)

”We followed their recommendations but maybe we used better protection that they said was needed, but I thought that the employees need to choose – if they want a hat on they could have a hat on if we have them” (Unit manager, geriatrics inpatient care, case A) .

“If you want an honest answer, it is well…. But if we shall follow the authorities its absolutely yes. Who will say that we should stop using it?” (Chief of nurse emergency department, case A).

“ICU got everything and then the other personal got normal masks.” (Manager internal medicine, case A).

"Everyone was really scared, just like with these patients, so it was a very chaotic start, you could say" (Unit manager inpatient care, Case A) .

”But we, we were never in a situation where, you know, we ran out of supplies on weekends, or any day for that matter. If that happened, we could always call the supply department, and they would arrange the supplies for us. They always had stock, and someone was in charge of it. It wasn't like the usual situation where you place an order, and then it arrives.” (Chief nurse, emergency department ,case A).

”Everyone started hoarding hand sanitizer. We had hand sanitizer, you know. But it started disappearing too, as it became a target for theft. We had to lock it up, put extra locks on doors, issue masks, and things like that.” (Property manager, case A).

"All medications were in short supply. We still have medications that were purchased during the pandemic. Our pharmacy department was very good at determining what medications we needed and procuring as much as they could. And then, you know, there were Chinese medications, Russian medications, with no Swedish labels, written in Russian or Chinese. 'This is propofol that you should use.' Well, I had no idea what it said on them." (Unit manager, anesthesia, case A).

“Initially, there was a lot of anxiety about getting infected. People felt like they didn't have the best protective gear. Then, on social media, you'd see pictures from other facilities with these visors that looked better, and you'd wonder why we didn't have them.” (Department manager, Surgery, case A).

"It was all about protective gear, primarily, and that was the big concern initially." (Nursing manager, case A).

"There was a tremendous fear at the beginning, the fear that there wouldn't be enough for everyone. So, people almost started running to each other's departments and taking things - you understand, it was a bit like that. It was a bit tough at the beginning, and also, it was critical with supplies, so to speak. We had to think about how to acquire them. Should we buy raincoats? Yes, because at the beginning, we thought, 'Now we have to do this so that we can disinfect because we don't have any.' But it never came to that." (Unit manager inpatient care, case A).

"There was a lot of work, especially last spring when there was a panic about protective gear." (leader of the supply group, case A).

"For our part, waves two and three have been much calmer, really.” (leader of the supply group, case A).

"Asked for the same protective gear as those in the ICU." (Service manager case A).

"Yes, the protective gear we initially received was a bit strange." (Chief nurse emergency department, case A).

"Yes, and it happened quite early. When the respirators and such were running out, we understood that... that's how it began. Disposable materials ran out very early. From my standpoint, that was probably the worst. I had a sinking feeling in my stomach, thinking, 'Are we standing here without respirators?' That's when we had to start looking for reusable solutions. When disposable equipment wasn't available, how could we find something that could last year after year? So, we turned to the industry for that." (Business developer at IVA , case A).

"And there was nothing. It created frustration among all the staff. We were supposed to work with this deadly and society-threatening disease and not have enough protection or not even know what would be sufficient protection. It created a lot of frustration, fear, and anxiety among the staff, where you might..." (CEO secretary, case A).

"So, we had night shifts where we received deliveries. During that night shift, worried staff could call and place orders and such. It was for a relatively short period, maybe two to three weeks, but it was very exhausting for a few people working. R: So, you changed it to round-the-clock staffing on the supply side. One of our strategies to provide reassurance and maintain a dialogue, so that it was never closed. R: How many of you shared this duty? Well, with a bit of hindsight, there were actually only two of us. R: So, you worked in shifts then? Yes, yes, we did." (Service manager, case A).

“And it was very difficult until we actually changed our approach. Yes, we did that in April last year. Because for me, logistics is like... either you have a pull logistics system where the customer places orders and we produce based on demand. But that didn't really work here because if they just ordered and we didn't have enough, we would constantly run out. So, we changed our approach to what's called push logistics, where we monitored or inventoried their protective gear and replenished it according to the calculated average, so to speak. And then, things started to calm down in healthcare. But if we let them order big max from us that we didn't have, it was very stressful. “(Service manager, case A).

"So, we were there, thinking we can't have it like this. Because the healthcare staff would call this supply group at night because they were worried. As soon as something ran out, and all it took was for someone to have used it up or some poor soul took things home out of fear. Yes, probably a lot of that happened. I can't pinpoint anything, but there was excessive use of protective gear.”(Service manager, case A).

"It was very early, but, as I said, I joke a bit that I hit the wall two or three times during the spring, and that's how I experienced it. Because, as I said, the hospital is building up functions. The CEO is working on it, but it's not about that. It's about the people. So, we had night shifts where we received deliveries, and during that night shift, worried staff could call and place orders and such. It was for a relatively short period, maybe two to three weeks, but it was very exhausting for a few people working." (Service manager, case A).

“R: So, you changed it to round-the-clock staffing on the supply side.

A: One of our strategies to provide reassurance and maintain a dialogue, so that it was never closed.

R: How many of you shared this duty?

A: Well, with a bit of hindsight, there were actually only two of us.

R: So, you worked in shifts then?

A: Yes, yes, we did." (Service manager case A)

R: And that was what you planned for? During the summer, after the first wave?

A: Actually, it was towards the end of the first wave when we found... now we dare to do it, and our delivery reliability improved so much that we actually dared to set up these buffer forecasts or buffer levels. Then it was very easy to run the logistics. You just keep subtracting from the buffer level when you make deliveries, and then you keep replenishing up to the buffer limit all the time." (Service manager, case A).

“Exactly, a procurement... all our crisis purchases and procurement functions, they combined and moved into some kind of unit at the university hospital – I actually visited there. At a hospital, and I conducted a quick situation analysis, from what I understood. I realized we didn't have the network or the expertise to get these things that we needed, so they asked for help and got, among other things, laid-off Scania buyers with their network – so it was a fantastic match made in heaven, I would say. And then they started creating with almost no procurement limits – it must have been fantastic to work there.” (Service manager, case A).

“Until we got forecasts, and they worked very lean in that way. They, after a while of nagging, actually made visible what was on its way, what our expected balance was, and how much each care provider should receive according to a calculation model that we also questioned and had to redo. And we insisted on approximately what we were consuming. So, we managed to establish a quite efficient supply flow, actually, when it started to come together." (Service manager, case A).

“So, we had night shifts where we received deliveries, and on those night shifts, anxious staff could call and place orders and such. It was during a fairly short period - maybe for two or three weeks, but it was very exhausting for a few individuals to work.” (Service manager, case A).

“When the pandemic hit, we switched gears very quickly. Initially, we had a lot of training on personal protective equipment and how to handle this infection.” (nursing manager, case A).

“Yes, we had protective equipment for the most part. Hand sanitizer ran out. Then aprons and masks also ran out before we received a dispensation from the National Board of Health and Welfare for the FFP3 masks. ‘(Department manager, emergency department, case B).
