# Supplementary material for: Managing disrupted supply chains in Swedish hospitals during the COVID-19 pandemic
Source: Health Syst (Basingstoke). 2024 May 7;14(1):58–68. doi: 10.1080/20476965.2024.2349816 (PMC11843631; doi:10.1080/20476965.2024.2349816)
Supplement: Supplemental Material [file THSS_A_2349816_SM1633.zip › Inteview guide in swedish.docx]

# Interview guide

Research questions:

Vilka element krävs för att skapa rätt kapacitet under en pandemi?

How is the organization and management changing with respect to hierarchy during Covid-19 (higher demand)? Are there elements in the crisis management that would be advantageous to keep during normal demand?

## Questions:

**Intervjuperson:**

Namn

Position

Hur länge har du varit på sjukhuset och i din nuvarande position. Kan du berätta lite om din bakgrund?

**Krisens storlek och omfattning (siffror tar jag också fram så det är inte det som är frågan):**

1. Vilken känsla har du av krisens storlek under första vågen under mars-maj?
2. Vilken känsla har du av krisens storlek just nu?

**Krisberedskapsplan och organisation (dokument införskaffas):**

1. Har ni någon krisberedskapsplan?
2. Är planen inriktad på olyckor eller pandemi eller hanterar planen olika typer av krisberedskap?
3. Har ni rutiner för uppdatering av planen?
4. Har planen uppdaterats under pandemin?
5. Preciserar er plan specifik kapacitetsuppbyggnad av vårdplatser och intensivvårdsplatser?
6. Hur har ni förändrat er organisation enligt plan eller ej enligt plan under Covid-19? Har ni tex haft en speciell krisledning och vem har den då bestått av?

**Uppbyggnad av kapacitet (siffror tages från systemen)**

1. Om ni hade specificerad kapacitetsuppbyggnad i beredskapsplanen – matchades den i verkligheten?
2. Var det balans mellan kapacitet och behov för covid-19 patienterna /tidsenhet? Under första vågen (mars- maj) och under andra vågen (nov-?)?
3. Har uppbyggnaden av kapaciteten (provtagning/vårdplatser/IVA platser/akuten/andra av er upptäckta viktiga kapaciteter hädanefter kallad kapaciteter) gjorts efter hand efter behovet eller utefter prognoser. Under första vågen (mars- maj) och under andra vågen (nov-?)
4. Om uppbyggnaden gjorts utefter prognoser – vem (nationell, regional, lokal) och om lokalt hur gjorde ni prognosen?
5. Hur har ni hanterat neddragningar/återuppbyggnad av kapacitet för annan vård? Har all befintlig kapacitet för annan vård utnyttjats? Under första vågen (mars- maj) och under andra vågen (nov-?)?
6. Hur har kapacitet och patientflöden förändrats för att förhindra att Covid-19 patienterna blandas med andra patienter på vårdavdelningar/akuten.
7. Har den sänkning i vårdtid framförallt på IVA något med brist på kapacitet att göra eller är det en ren kunskapshöjning?
8. Har ni anställt fler medarbetare?
9. Har ni flyttat runt medarbetare?
10. Har ni ändrat skiftformer/ använt övertid?
11. Har belastningen per medarbetare varit jämn över sjukhuset?
12. Vad har ni gjort för att er personal skall orka igenom pandemin?
13. Vilka element var enligt dig viktigast under uppbyggnaden av rätt kapacitet?

**Ledarskap (dokument om instruktioner från nationellt och regionalt håll införskaffas):**

1. Vilka instruktioner/styrning kring organisation och kapacitetsuppbyggnad menar du att ni har ni fått från nationellt håll: Regering, Folkhälsomyndigheter, socialstyrelse och SKR?
2. Hur har detta tagits emot och använts i er egen organisation?
3. Vem i er organisation har tagit emot och verkställt informationen från nationell nivå?
4. Vilka instruktioner/styrning kring organisation och kapacitetsuppbyggnad har ni fått från politisk ledning på regional nivå och regionledning och hur har detta använts enligt dig?
5. Hur såg/ser ledningen ut för sjukhuset under Covid -19? Är det någon skillnad mellan första och andra vågen?
6. På vilken beslutsnivå på sjukhuset togs beslut om storleken på kapacitetsuppbyggnaden och hur detta skulle ske?
7. Vilken data användes och på vilka grunder togs besluten?
8. Vem menar du tog ledarskapet (formellt eller informellt) under uppbyggnad av kapaciteten? Överensstämde denna ledning med den beskriven krisberedskapsplanen?
9. Hur har du upplevt att ledarskapet har förändrats hos er under Covid -19? (mer eller mindre hierarkiskt /mer eller mindre centraliserat/detaljerat eller på annat sätt)
10. Har ledningen/ledarskapet fungerat bra/mindre bra under pandemin jämfört med normalläge?
11. Har du någon idé om vad som kunde varit – kan bli bättre i ledarskapet under Covid-19? (mer eller mindre hierarkiskt /mer eller mindre centraliserat/detaljerat eller på annat sätt)
12. Kan något av den kunskap om hur ni använt organisation och ledarskap under pandemin användas för att förbättra det ordinarie ledarskapet? (mer eller mindre hierarkiskt /mer eller mindre centraliserat/detaljerat eller på annat sätt)?
13. Vilka delar i ledarskapet var enligt dig viktigast för att skapa möjlighet att bygga rätt kapacitet?

**Informationsflöde**

1. Hur har informations och kunskapsflödet kring prognostiserat kapacitetsbehov utifrån senaste fakta fungerat?
2. Vem/var/hur ofta uppdatering?
3. Hur har det medicinska informations och kunskapsflödet fungerat kring behandlingsmetoder (med tanke på en halvering av IVA vårdtiden mellan första och andra vågen)? Via nationella institutioner, via uppbyggda nationella nätverk, via ledningen på sjukhuset, via kollegiala kontakter, forskningsartiklar eller på annat sätt?
4. Hur ofta har ni uppdaterat er på senaste rön?
5. Hur har informationsflödet påverkat möjligheten att bygga rätt kapacitet.
