# Supplementary material for: Managing disrupted supply chains in Swedish hospitals during the COVID-19 pandemic
Source: Health Syst (Basingstoke). 2024 May 7;14(1):58–68. doi: 10.1080/20476965.2024.2349816 (PMC11843631; doi:10.1080/20476965.2024.2349816)
Supplement: Supplemental Material [file THSS_A_2349816_SM1633.zip › MR_ICU during first wave.pdf]

```
REGRESSION
  /MISSING LISTWISE
  /STATISTICS COEFF OUTS R ANOVA COLLIN TOL
  /CRITERIA=PIN(.05) POUT(.10)
  /NOORIGIN
  /DEPENDENT @68a
  /METHOD=BACKWARD @33a @34a @35a @37a @38a @39a.
```

Regression

Model Summary

Model	R	R Square	Adjusted R Square	Std. Error of the Estimate
1	,354[a]	,125	,049	1,307
2	,353[b]	,125	,062	1,298
3	,351[c]	,123	,074	1,289
4	,349[d]	,122	,085	1,282
5	,344[e]	,118	,094	1,276
6	,340[f]	,116	,104	1,269

a. Predictors: (Constant), 6, 3, 2, 1, 5, 4

b. Predictors: (Constant), 6, 3, 2, 1, 4

c. Predictors: (Constant), 6, 3, 2, 1

d. Predictors: (Constant), 6, 2, 1

e. Predictors: (Constant), 6, 1

f. Predictors: (Constant), 1

ANOVA[a]

Model		Sum of Squares	df	Mean Square	F	Sig.
1	Regression	16,891	6	2,815	1,649	,147[b]
	Residual	117,780	69	1,707		
	Total	134,671	75			
2	Regression	16,788	5	3,358	1,994	,090[c]
	Residual	117,883	70	1,684		
	Total	134,671	75			
3	Regression	16,630	4	4,157	2,501	,050[d]
	Residual	118,041	71	1,663		
	Total	134,671	75			
4	Regression	16,387	3	5,462	3,325	,024[e]
	Residual	118,284	72	1,643		
	Total	134,671	75			
5	Regression	15,899	2	7,949	4,886	,010[f]
	Residual	118,772	73	1,627		
	Total	134,671	75			
6	Regression	15,556	1	15,556	9,664	,003[g]
	Residual	119,115	74	1,610		
	Total	134,671	75			

a. Dependent Variable: 13

b. Predictors: (Constant), 6, 3, 2, 1, 5, 4

c. Predictors: (Constant), 6, 3, 2, 1, 4

d. Predictors: (Constant), 6, 3, 2, 1

e. Predictors: (Constant), 6, 2, 1

f. Predictors: (Constant), 6, 1

g. Predictors: (Constant), 1

Coefficients[a]

Model		Unstandardized Coefficients		Standardized Coefficients	t	Sig.	Collinearity Statistics
		B	Std. Error	Beta			Tolerance
1	(Constant)	4,433	,595		7,454	,000	
	1	-,336	,146	-,312	-2,293	,025	,685
	2	,053	,108	,060	,490	,625	,858
	3	-,076	,208	-,042	-,365	,716	,946
	4	,074	,190	,069	,387	,700	,395
	5	-,034	,138	-,038	-,246	,806	,518
	6	-,100	,159	-,096	-,625	,534	,542
2	(Constant)	4,438	,590		7,517	,000	
	1	-,334	,145	-,310	-2,299	,025	,687
	2	,048	,105	,054	,455	,651	,890
	3	-,080	,206	-,045	-,390	,698	,953
	4	,050	,163	,047	,306	,760	,534
	6	-,103	,158	-,099	-,655	,514	,547
3	(Constant)	4,477	,573		7,816	,000	
	1	-,323	,140	-,300	-2,311	,024	,734
	2	,055	,103	,061	,533	,595	,930
	3	-,078	,205	-,044	-,382	,703	,954
	6	-,080	,136	-,076	-,583	,561	,719
4	(Constant)	4,409	,541		8,151	,000	
	1	-,333	,136	-,309	-2,451	,017	,765
	2	,056	,102	,062	,545	,587	,931
	6	-,078	,136	-,075	-,577	,566	,719
5	(Constant)	4,512	,504		8,955	,000	
	1	-,336	,135	-,312	-2,484	,015	,766
	6	-,060	,131	-,058	-,459	,648	,766
6	(Constant)	4,420	,459		9,626	,000	
	1	-,366	,118	-,340	-3,109	,003	1,000

Coefficients[a]

Model		Collinearity Statistics VIF
1	(Constant)	
	1	1,459
	2	1,166
	3	1,057
	4	2,531
	5	1,931
	6	1,846
2	(Constant)	
	1	1,455
	2	1,123
	3	1,049
	4	1,873
	6	1,829
3	(Constant)	
	1	1,362
	2	1,075
	3	1,048
	6	1,391
4	(Constant)	
	1	1,307
	2	1,074
	6	1,390
5	(Constant)	
	1	1,306
	6	1,306
6	(Constant)	
	1	1,000

a. Dependent Variable: 13

Excluded Variables[a]

Model		Beta In	t	Sig.	Partial Correlation	Collinearity Statistics	
						Tolerance	VIF
2	5	-,038[b]	-,246	,806	-,030	,518	1,931
3	5	-,008[c]	-,057	,955	-,007	,700	1,429
	4	,047[c]	,306	,760	,037	,534	1,873
4	5	-,012[d]	-,091	,927	-,011	,706	1,417
	4	,045[d]	,295	,769	,035	,535	1,870
	3	-,044[d]	-,382	,703	-,045	,954	1,048
5	5	,007[e]	,056	,955	,007	,760	1,316
	4	,060[e]	,403	,688	,047	,559	1,790
	3	-,045[e]	-,396	,693	-,047	,954	1,048
	2	,062[e]	,545	,587	,064	,931	1,074
6	5	-,015[f]	-,133	,894	-,016	,908	1,101
	4	,013[f]	,103	,918	,012	,772	1,295
	3	-,043[f]	-,383	,703	-,045	,956	1,047
	2	,046[f]	,418	,677	,049	,991	1,009
	6	-,058[f]	-,459	,648	-,054	,766	1,306

Excluded Variables[a]

Model		Collinearity ...
		Minimum Tolerance
2	5	,395
3	5	,628
	4	,534
4	5	,630
	4	,535
	3	,719
5	5	,641
	4	,554
	3	,735
	2	,719
6	5	,908
	4	,772
	3	,956
	2	,991
	6	,766

a. Dependent Variable: 13

b. Predictors in the Model: (Constant), 6, 3, 2, 1, 4

c. Predictors in the Model: (Constant), 6, 3, 2, 1

d. Predictors in the Model: (Constant), 6, 2, 1

e. Predictors in the Model: (Constant), 6, 1

f. Predictors in the Model: (Constant), 1
