# Supplementary material for: Managing disrupted supply chains in Swedish hospitals during the COVID-19 pandemic
Source: Health Syst (Basingstoke). 2024 May 7;14(1):58–68. doi: 10.1080/20476965.2024.2349816 (PMC11843631; doi:10.1080/20476965.2024.2349816)
Supplement: Supplemental Material [file THSS_A_2349816_SM1633.zip › MR_ICU during later waves.pdf]

```
REGRESSION
  /MISSING LISTWISE
  /STATISTICS COEFF OUTS R ANOVA COLLIN TOL
  /CRITERIA=PIN(.05) POUT(.10)
  /NOORIGIN
  /DEPENDENT @68b
  /METHOD=BACKWARD @33b @34b @35b @37b @38b @39b.
```

# Regression

**Model Summary**

| Model | R | R Square | Adjusted R Square | Std. Error of the Estimate |
|---|---|---|---|---|
| 1 | ,496[a] | ,246 | ,178 | ,878 |
| 2 | ,496[b] | ,246 | ,190 | ,872 |
| 3 | ,493[c] | ,243 | ,198 | ,867 |
| 4 | ,491[d] | ,241 | ,208 | ,862 |
| 5 | ,481[e] | ,232 | ,210 | ,861 |
| 6 | ,463[f] | ,214 | ,203 | ,864 |

a. Predictors: (Constant), 12, 9, 8, 7, 11, 10

b. Predictors: (Constant), 12, 9, 7, 11, 10

c. Predictors: (Constant), 12, 9, 7, 11

d. Predictors: (Constant), 12, 9, 11

e. Predictors: (Constant), 12, 11

f. Predictors: (Constant), 12

**ANOVA[a]**

| Model | | Sum of Squares | df | Mean Square | F | Sig. |
|---|---|---|---|---|---|---|
| 1 | Regression | 16,610 | 6 | 2,768 | 3,590 | ,004[b] |
| | Residual | 50,897 | 66 | ,771 | | |
| | Total | 67,507 | 72 | | | |
| 2 | Regression | 16,599 | 5 | 3,320 | 4,369 | ,002[c] |
| | Residual | 50,908 | 67 | ,760 | | |
| | Total | 67,507 | 72 | | | |
| 3 | Regression | 16,393 | 4 | 4,098 | 5,452 | ,001[d] |
| | Residual | 51,114 | 68 | ,752 | | |
| | Total | 67,507 | 72 | | | |
| 4 | Regression | 16,244 | 3 | 5,415 | 7,288 | ,000[e] |
| | Residual | 51,263 | 69 | ,743 | | |
| | Total | 67,507 | 72 | | | |
| 5 | Regression | 15,635 | 2 | 7,818 | 10,550 | ,000[f] |
| | Residual | 51,871 | 70 | ,741 | | |
| | Total | 67,507 | 72 | | | |
| 6 | Regression | 14,464 | 1 | 14,464 | 19,360 | ,000[g] |
| | Residual | 53,043 | 71 | ,747 | | |
| | Total | 67,507 | 72 | | | |

a. Dependent Variable: 14

b. Predictors: (Constant), 12, 9, 8, 7, 11, 10

c. Predictors: (Constant), 12, 9, 7, 11, 10

d. Predictors: (Constant), 12, 9, 7, 11

e. Predictors: (Constant), 12, 9, 11

f. Predictors: (Constant), 12, 11

g. Predictors: (Constant), 12

**Coefficients[a]**

| Model | | Unstandardized Coefficients | | Standardized Coefficients | t | Sig. | Collinearity Statistics |
|---|---|---|---|---|---|---|---|
| | | B | Std. Error | Beta | | | Tolerance |
| 1 | (Constant) | 4,929 | ,530 | | 9,306 | ,000 | |
| | 7 | -,068 | ,131 | -,059 | -,523 | ,603 | ,902 |
| | 8 | -,010 | ,086 | -,013 | -,119 | ,906 | ,963 |
| | 9 | ,333 | ,353 | ,104 | ,942 | ,349 | ,930 |
| | 10 | ,081 | ,159 | ,072 | ,509 | ,613 | ,572 |
| | 11 | -,233 | ,163 | -,190 | -1,430 | ,158 | ,647 |
| | 12 | -,518 | ,159 | -,412 | -3,249 | ,002 | ,710 |
| 2 | (Constant) | 4,906 | ,487 | | 10,080 | ,000 | |
| | 7 | -,070 | ,129 | -,061 | -,546 | ,587 | ,915 |
| | 9 | ,335 | ,350 | ,105 | ,957 | ,342 | ,933 |
| | 10 | ,082 | ,157 | ,073 | ,520 | ,605 | ,574 |
| | 11 | -,235 | ,161 | -,192 | -1,463 | ,148 | ,655 |
| | 12 | -,516 | ,157 | -,410 | -3,285 | ,002 | ,721 |
| 3 | (Constant) | 4,970 | ,468 | | 10,617 | ,000 | |
| | 7 | -,056 | ,125 | -,048 | -,445 | ,658 | ,961 |
| | 9 | ,302 | ,342 | ,095 | ,882 | ,381 | ,964 |
| | 11 | -,198 | ,143 | -,162 | -1,382 | ,172 | ,814 |
| | 12 | -,488 | ,147 | -,388 | -3,320 | ,001 | ,814 |
| 4 | (Constant) | 4,899 | ,437 | | 11,198 | ,000 | |
| | 9 | ,308 | ,340 | ,097 | ,905 | ,369 | ,966 |
| | 11 | -,200 | ,142 | -,163 | -1,402 | ,166 | ,814 |
| | 12 | -,499 | ,144 | -,397 | -3,462 | ,001 | ,837 |
| 5 | (Constant) | 5,207 | ,274 | | 19,008 | ,000 | |
| | 11 | -,176 | ,140 | -,144 | -1,258 | ,213 | ,843 |
| | 12 | -,510 | ,143 | -,406 | -3,558 | ,001 | ,843 |
| 6 | (Constant) | 5,038 | ,240 | | 21,018 | ,000 | |
| | 12 | -,582 | ,132 | -,463 | -4,400 | ,000 | 1,000 |

**Coefficients[a]**

| Model | | Collinearity Statistics VIF |
|---|---|---|
| 1 | (Constant) | |
| | 7 | 1,109 |
| | 8 | 1,039 |
| | 9 | 1,075 |
| | 10 | 1,749 |
| | 11 | 1,546 |
| | 12 | 1,409 |
| 2 | (Constant) | |
| | 7 | 1,092 |
| | 9 | 1,072 |
| | 10 | 1,743 |
| | 11 | 1,528 |
| | 12 | 1,386 |
| 3 | (Constant) | |
| | 7 | 1,040 |
| | 9 | 1,037 |
| | 11 | 1,229 |
| | 12 | 1,229 |
| 4 | (Constant) | |
| | 9 | 1,036 |
| | 11 | 1,228 |
| | 12 | 1,195 |
| 5 | (Constant) | |
| | 11 | 1,186 |
| | 12 | 1,186 |
| 6 | (Constant) | |
| | 12 | 1,000 |

a. Dependent Variable: 14

**Excluded Variables**[a]

| Model | | Beta In | t | Sig. | Partial Correlation | Collinearity Statistics | |
|---|---|---|---|---|---|---|---|
| | | | | | | Tolerance | VIF |
| 2 | 8 | -,013[b] | -,119 | ,906 | -,015 | ,963 | 1,039 |
| 3 | 8 | -,016[c] | -,150 | ,881 | -,018 | ,966 | 1,035 |
| | 10 | ,073[c] | ,520 | ,605 | ,063 | ,574 | 1,743 |
| 4 | 8 | -,021[d] | -,199 | ,843 | -,024 | ,978 | 1,022 |
| | 10 | ,056[d] | ,413 | ,681 | ,050 | ,602 | 1,660 |
| | 7 | -,048[d] | -,445 | ,658 | -,054 | ,961 | 1,040 |
| 5 | 8 | -,026[e] | -,240 | ,811 | -,029 | ,980 | 1,020 |
| | 10 | ,032[e] | ,240 | ,811 | ,029 | ,624 | 1,603 |
| | 7 | -,052[e] | -,481 | ,632 | -,058 | ,963 | 1,039 |
| | 9 | ,097[e] | ,905 | ,369 | ,108 | ,966 | 1,036 |
| 6 | 8 | -,037[f] | -,349 | ,728 | -,042 | ,988 | 1,012 |
| | 10 | -,036[f] | -,295 | ,769 | -,035 | ,751 | 1,332 |
| | 7 | -,054[f] | -,502 | ,617 | -,060 | ,963 | 1,038 |
| | 9 | ,069[f] | ,653 | ,516 | ,078 | 1,000 | 1,000 |
| | 11 | -,144[f] | -1,258 | ,213 | -,149 | ,843 | 1,186 |

**Excluded Variables**[a]

| Model | | Collinearity ... |
|---|---|---|
| | | Minimum Tolerance |
| 2 | 8 | ,572 |
| 3 | 8 | ,794 |
| | 10 | ,574 |
| 4 | 8 | ,807 |
| | 10 | ,602 |
| | 7 | ,814 |
| 5 | 8 | ,827 |
| | 10 | ,624 |
| | 7 | ,819 |
| | 9 | ,814 |
| 6 | 8 | ,988 |
| | 10 | ,751 |
| | 7 | ,963 |
| | 9 | 1,000 |
| | 11 | ,843 |

a. Dependent Variable: 14

b. Predictors in the Model: (Constant), 12, 9, 7, 11, 10

c. Predictors in the Model: (Constant), 12, 9, 7, 11

d. Predictors in the Model: (Constant), 12, 9, 11

e. Predictors in the Model: (Constant), 12, 11

f. Predictors in the Model: (Constant), 12
