# Supplementary material for: Managing disrupted supply chains in Swedish hospitals during the COVID-19 pandemic
Source: Health Syst (Basingstoke). 2024 May 7;14(1):58–68. doi: 10.1080/20476965.2024.2349816 (PMC11843631; doi:10.1080/20476965.2024.2349816)
Supplement: Supplemental Material [file THSS_A_2349816_SM1633.zip › MR_other units during first wave.pdf]

```
REGRESSION
  /MISSING LISTWISE
  /STATISTICS COEFF OUTS R ANOVA COLLIN TOL
  /CRITERIA=PIN(.05) POUT(.10)
  /NOORIGIN
  /DEPENDENT @68a
  /METHOD=BACKWARD @33a @34a @35a @37a @38a @39a.
```

Regression

Model Summary

Model	R	R Square	Adjusted R Square	Std. Error of the Estimate
1	,458[a]	,210	,174	1,116
2	,456[b]	,208	,178	1,114
3	,452[c]	,204	,180	1,112
4	,448[d]	,201	,183	1,110
5	,437[e]	,191	,179	1,113

a. Predictors: (Constant), 6, 3, 2, 1, 5, 4

b. Predictors: (Constant), 6, 3, 1, 5, 4

c. Predictors: (Constant), 6, 3, 1, 4

d. Predictors: (Constant), 6, 3, 1

e. Predictors: (Constant), 3, 1

ANOVA[a]

Model		Sum of Squares	df	Mean Square	F	Sig.
1	Regression	43,098	6	7,183	5,764	,000[b]
	Residual	162,012	130	1,246		
	Total	205,109	136			
2	Regression	42,663	5	8,533	6,881	,000[c]
	Residual	162,446	131	1,240		
	Total	205,109	136			
3	Regression	41,909	4	10,477	8,474	,000[d]
	Residual	163,201	132	1,236		
	Total	205,109	136			
4	Regression	41,139	3	13,713	11,123	,000[e]
	Residual	163,971	133	1,233		
	Total	205,109	136			
5	Regression	39,135	2	19,567	15,798	,000[f]
	Residual	165,975	134	1,239		
	Total	205,109	136			

a. Dependent Variable: 13

b. Predictors: (Constant), 6, 3, 2, 1, 5, 4

c. Predictors: (Constant), 6, 3, 1, 5, 4

d. Predictors: (Constant), 6, 3, 1, 4

e. Predictors: (Constant), 6, 3, 1

f. Predictors: (Constant), 3, 1

Coefficients[a]

Model		Unstandardized Coefficients		Standardized Coefficients	t	Sig.	Collinearity Statistics
		B	Std. Error	Beta			Tolerance
1	(Constant)	4,813	,320		15,019	,000	
	1	-,118	,091	-,138	-1,289	,200	,528
	2	-,043	,073	-,048	-,590	,556	,921
	3	-,370	,098	-,333	-3,755	,000	,774
	4	-,103	,112	-,105	-,918	,360	,465
	5	,084	,098	,089	,858	,392	,567
	6	-,115	,103	-,113	-1,124	,263	,598
2	(Constant)	4,727	,285		16,613	,000	
	1	-,117	,091	-,137	-1,282	,202	,528
	3	-,365	,098	-,328	-3,728	,000	,779
	4	-,109	,111	-,112	-,984	,327	,470
	5	,076	,097	,080	,780	,437	,580
	6	-,115	,102	-,113	-1,127	,262	,598
3	(Constant)	4,755	,282		16,871	,000	
	1	-,115	,091	-,135	-1,268	,207	,528
	3	-,359	,097	-,323	-3,683	,000	,784
	4	-,084	,106	-,085	-,789	,431	,515
	6	-,083	,093	-,081	-,886	,377	,720
4	(Constant)	4,692	,270		17,380	,000	
	1	-,153	,078	-,179	-1,963	,052	,722
	3	-,364	,097	-,328	-3,758	,000	,789
	6	-,110	,086	-,108	-1,275	,205	,837
5	(Constant)	4,542	,243		18,653	,000	
	1	-,191	,072	-,224	-2,663	,009	,851
	3	-,331	,094	-,298	-3,537	,001	,851

Coefficients[a]

Model		Collinearity Statistics VIF
1	(Constant)	
	1	1,894
	2	1,086
	3	1,292
	4	2,148
	5	1,764
	6	1,671
2	(Constant)	
	1	1,893
	3	1,283
	4	2,127
	5	1,724
	6	1,671
3	(Constant)	
	1	1,893
	3	1,275
	4	1,941
	6	1,389
4	(Constant)	
	1	1,385
	3	1,268
	6	1,195
5	(Constant)	
	1	1,175
	3	1,175

a. Dependent Variable: 13

Excluded Variables[a]

Model		Beta In	t	Sig.	Partial Correlation	Collinearity Statistics	
						Tolerance	VIF
2	2	-,048[b]	-,590	,556	-,052	,921	1,086
3	2	-,037[c]	-,467	,641	-,041	,942	1,061
	5	,080[c]	,780	,437	,068	,580	1,724
4	2	-,046[d]	-,579	,564	-,050	,963	1,038
	5	,050[d]	,512	,610	,045	,636	1,573
	4	-,085[d]	-,789	,431	-,069	,515	1,941
5	2	-,058[e]	-,740	,461	-,064	,980	1,020
	5	-,017[e]	-,198	,843	-,017	,852	1,174
	4	-,121[e]	-1,209	,229	-,104	,599	1,670
	6	-,108[e]	-1,275	,205	-,110	,837	1,195

Excluded Variables[a]

Model		Collinearity ...
		Minimum Tolerance
2	2	,465
3	2	,504
	5	,470
4	2	,718
	5	,624
	4	,515
5	2	,836
	5	,733
	4	,536
	6	,722

a. Dependent Variable: 13

b. Predictors in the Model: (Constant), 6, 3, 1, 5, 4

c. Predictors in the Model: (Constant), 6, 3, 1, 4

d. Predictors in the Model: (Constant), 6, 3, 1

e. Predictors in the Model: (Constant), 3, 1
