# Supplementary material for: Managing disrupted supply chains in Swedish hospitals during the COVID-19 pandemic
Source: Health Syst (Basingstoke). 2024 May 7;14(1):58–68. doi: 10.1080/20476965.2024.2349816 (PMC11843631; doi:10.1080/20476965.2024.2349816)
Supplement: Supplemental Material [file THSS_A_2349816_SM1633.zip › MR_other units during later waves.pdf]

```
REGRESSION
  /MISSING LISTWISE
  /STATISTICS COEFF OUTS R ANOVA COLLIN TOL
  /CRITERIA=PIN(.05) POUT(.10)
  /NOORIGIN
  /DEPENDENT @68b
  /METHOD=BACKWARD @33b @34b @35b @37b @38b @39b.
```

Regression

Model Summary

Model	R	R Square	Adjusted R Square	Std. Error of the Estimate
1	,527[a]	,277	,244	,773
2	,527[b]	,277	,250	,770
3	,525[c]	,275	,253	,769
4	,510[d]	,260	,244	,773

a. Predictors: (Constant), 12, 8, 9, 10, 7, 11

b. Predictors: (Constant), 12, 8, 9, 10, 7

c. Predictors: (Constant), 12, 9, 10, 7

d. Predictors: (Constant), 9, 10, 7

ANOVA[a]

Model		Sum of Squares	df	Mean Square	F	Sig.
1	Regression	29,617	6	4,936	8,254	,000[b]
	Residual	77,141	129	,598		
	Total	106,757	135			
2	Regression	29,616	5	5,923	9,982	,000[c]
	Residual	77,142	130	,593		
	Total	106,757	135			
3	Regression	29,375	4	7,344	12,432	,000[d]
	Residual	77,382	131	,591		
	Total	106,757	135			
4	Regression	27,803	3	9,268	15,494	,000[e]
	Residual	78,954	132	,598		
	Total	106,757	135			

a. Dependent Variable: 14

b. Predictors: (Constant), 12, 8, 9, 10, 7, 11

c. Predictors: (Constant), 12, 8, 9, 10, 7

d. Predictors: (Constant), 12, 9, 10, 7

e. Predictors: (Constant), 9, 10, 7

Coefficients[a]

Model		Unstandardized Coefficients		Standardized Coefficients	t	Sig.	Collinearity Statistics
		B	Std. Error	Beta			Tolerance
1	(Constant)	5,470	,228		23,980	,000	
	7	-,273	,103	-,259	-2,658	,009	,591
	8	-,035	,055	-,050	-,635	,526	,922
	9	-,282	,141	-,154	-1,999	,048	,948
	10	-,309	,092	-,325	-3,376	,001	,606
	11	,004	,102	,004	,041	,968	,587
	12	,146	,103	,138	1,417	,159	,590
2	(Constant)	5,471	,225		24,355	,000	
	7	-,273	,102	-,259	-2,669	,009	,592
	8	-,035	,055	-,049	-,637	,525	,929
	9	-,282	,141	-,154	-2,007	,047	,949
	10	-,308	,089	-,324	-3,473	,001	,640
	12	,148	,090	,140	1,636	,104	,760
3	(Constant)	5,420	,209		25,930	,000	
	7	-,283	,101	-,269	-2,815	,006	,608
	9	-,283	,140	-,154	-2,020	,045	,949
	10	-,312	,088	-,328	-3,531	,001	,643
	12	,147	,090	,139	1,631	,105	,760
4	(Constant)	5,503	,204		26,995	,000	
	7	-,240	,098	-,227	-2,455	,015	,654
	9	-,263	,141	-,143	-1,872	,063	,956
	10	-,282	,087	-,296	-3,240	,002	,673

Coefficients[a]

Model		Collinearity Statistics VIF
1	(Constant)	
	7	1,692
	8	1,085
	9	1,054
	10	1,650
	11	1,702
	12	1,694
2	(Constant)	
	7	1,689
	8	1,077
	9	1,054
	10	1,562
	12	1,316
3	(Constant)	
	7	1,645
	9	1,054
	10	1,555
	12	1,315
4	(Constant)	
	7	1,529
	9	1,046
	10	1,485

a. Dependent Variable: 14

Excluded Variables[a]

Model		Beta In	t	Sig.	Partial Correlation	Collinearity Statistics	
						Tolerance	VIF
2	11	,004[b]	,041	,968	,004	,587	1,702
3	11	-,001[c]	-,015	,988	-,001	,592	1,689
	8	-,049[c]	-,637	,525	-,056	,929	1,077
4	11	,064[d]	,751	,454	,065	,762	1,312
	8	-,047[d]	-,610	,543	-,053	,929	1,076
	12	,139[d]	1,631	,105	,141	,760	1,315

Excluded Variables[a]

Model		Collinearity ...
		Minimum Tolerance
2	11	,587
3	11	,591
	8	,592
4	11	,610
	8	,635
	12	,608

a. Dependent Variable: 14

b. Predictors in the Model: (Constant), 12, 8, 9, 10, 7

c. Predictors in the Model: (Constant), 12, 9, 10, 7

d. Predictors in the Model: (Constant), 9, 10, 7
