# Supplementary material for: Managing disrupted supply chains in Swedish hospitals during the COVID-19 pandemic
Source: Health Syst (Basingstoke). 2024 May 7;14(1):58–68. doi: 10.1080/20476965.2024.2349816 (PMC11843631; doi:10.1080/20476965.2024.2349816)
Supplement: Supplemental Material [file THSS_A_2349816_SM1633.zip › PCA_ICU during first wave.pdf]

Factor Analysis

KMO and Bartlett's Test

Kaiser-Meyer-Olkin Measure of Sampling Adequacy.		,756
Bartlett's Test of Sphericity	Approx. Chi-Square	120,571
	df	15
	Sig.	,000

Communalities

	Initial	Extraction
1	1,000	,591
2	1,000	,507
3	1,000	,653
4	1,000	,772
5	1,000	,646
6	1,000	,646

Extraction Method: Principal Component Analysis.

Total Variance Explained

	Initial Eigenvalues			Extraction Sums of Squared Loadings		
Component	Total	% of Variance	Cumulative %	Total	% of Variance	Cumulative %
1	2,735	45,588	45,588	2,735	45,588	45,588
2	1,081	18,012	63,601	1,081	18,012	63,601
3	,852	14,196	77,797			
4	,620	10,325	88,122			
5	,436	7,271	95,393			
6	,276	4,607	100,000			

Total Variance Explained

	Rotation Sums of Squared Loadings		
Component	Total	% of Variance	Cumulative %
1	2,655	44,243	44,243
2	1,161	19,358	63,601
3			
4			
5			
6			

Extraction Method: Principal Component Analysis.

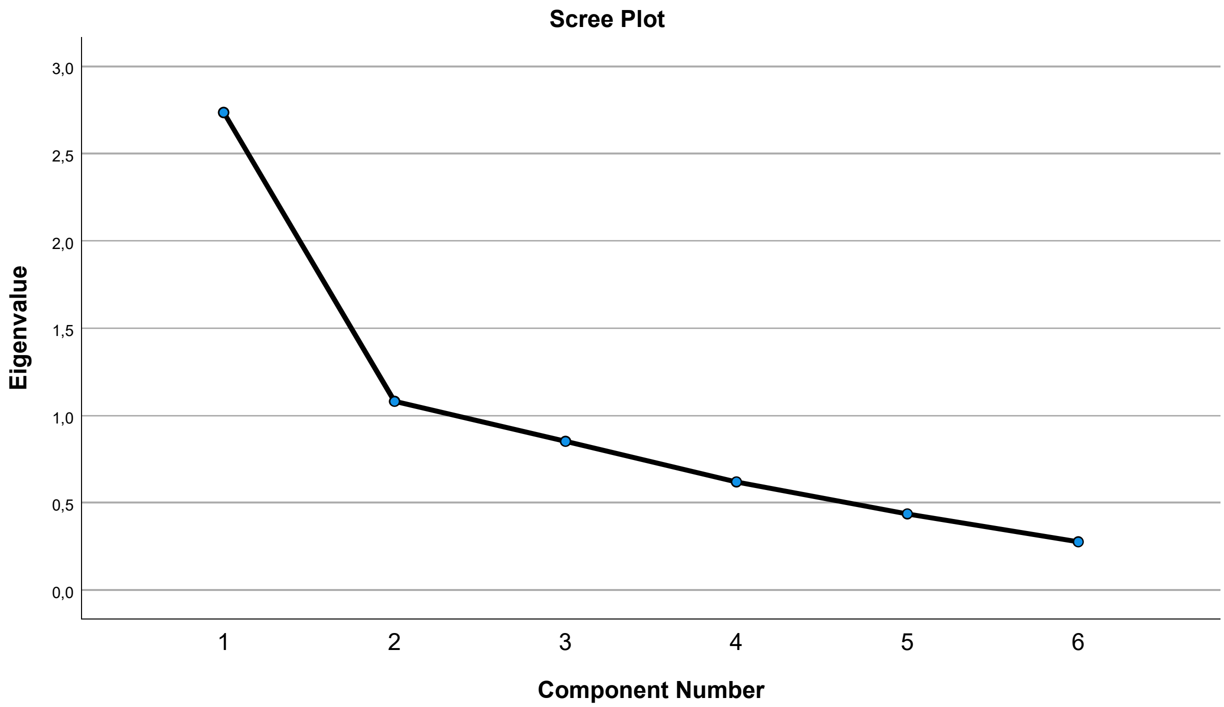

Component Matrix[a]

	Component	
	1	2
1	,656	,401
2	,470	-,535
3	,221	,777
4	,877	-,056
5	,788	-,160
6	,804	-,010

Extraction Method: Principal Component Analysis.

a. 2 components extracted.

Rotated Component Matrix[a]

	Component	
	1	2
1	,551	,536
2	,576	-,418
3	,043	,807
4	,868	,139
5	,804	,018
6	,786	,167

Extraction Method: Principal Component Analysis.
Rotation Method: Varimax with Kaiser Normalization.[a]

a. Rotation converged in 3 iterations.

Component Transformation Matrix

Component	1	2
1	,975	,221
2	-,221	,975

Extraction Method: Principal Component Analysis.
Rotation Method: Varimax with Kaiser Normalization.
