# Supplementary material for: Managing disrupted supply chains in Swedish hospitals during the COVID-19 pandemic
Source: Health Syst (Basingstoke). 2024 May 7;14(1):58–68. doi: 10.1080/20476965.2024.2349816 (PMC11843631; doi:10.1080/20476965.2024.2349816)
Supplement: Supplemental Material [file THSS_A_2349816_SM1633.zip › PCA_ICU during later waves.pdf]

Factor Analysis

KMO and Bartlett's Test

Kaiser-Meyer-Olkin Measure of Sampling Adequacy.		,745
Bartlett's Test of Sphericity	Approx. Chi-Square	176,018
	df	15
	Sig.	,000

Communalities

	Initial	Extraction
7	1,000	,605
8	1,000	,158
9	1,000	,099
10	1,000	,579
11	1,000	,583
12	1,000	,580

Extraction Method: Principal Component Analysis.

Total Variance Explained

	Initial Eigenvalues			Extraction Sums of Squared Loadings		
Component	Total	% of Variance	Cumulative %	Total	% of Variance	Cumulative %
1	2,604	43,401	43,401	2,604	43,401	43,401
2	,950	15,830	59,230			
3	,921	15,347	74,578			
4	,705	11,752	86,330			
5	,456	7,606	93,936			
6	,364	6,064	100,000			

Extraction Method: Principal Component Analysis.

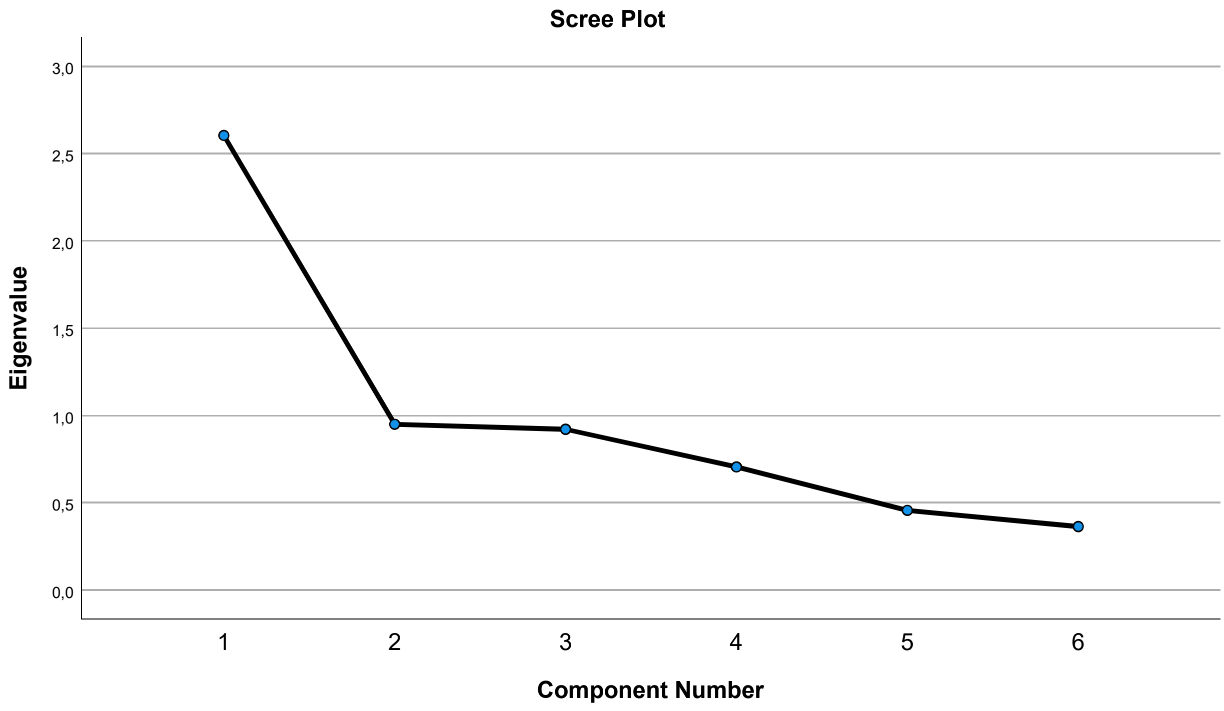

Component Matrix[a]

	Component
	1
7	,778
8	,397
9	,315
10	,761
11	,763
12	,762

Extraction Method:
Principal Component
Analysis.

a. 1 components extracted.

Rotated Component Matrix[a]

a. Only one component was extracted. The solution cannot be rotated.

Factor Analysis

KMO and Bartlett's Test

Kaiser-Meyer-Olkin Measure of Sampling Adequacy.		,613
Bartlett's Test of Sphericity	Approx. Chi-Square	60,178
	df	15
	Sig.	,000

Communalities

	Initial	Extraction
7	1,000	,469
8	1,000	,844
9	1,000	,845
10	1,000	,739
11	1,000	,694
12	1,000	,635

Extraction Method: Principal Component Analysis.

Total Variance Explained

Component	Initial Eigenvalues			Extraction Sums of Squared Loadings		
	Total	% of Variance	Cumulative %	Total	% of Variance	Cumulative %
1	2,068	34,467	34,467	2,068	34,467	34,467
2	1,111	18,521	52,988	1,111	18,521	52,988
3	1,047	17,446	70,434	1,047	17,446	70,434
4	,845	14,087	84,521			
5	,548	9,137	93,658			
6	,381	6,342	100,000			

Total Variance Explained

Component	Rotation Sums of Squared Loadings		
	Total	% of Variance	Cumulative %
1	2,053	34,223	34,223
2	1,088	18,135	52,358
3	1,085	18,076	70,434
4			
5			
6			

Extraction Method: Principal Component Analysis.

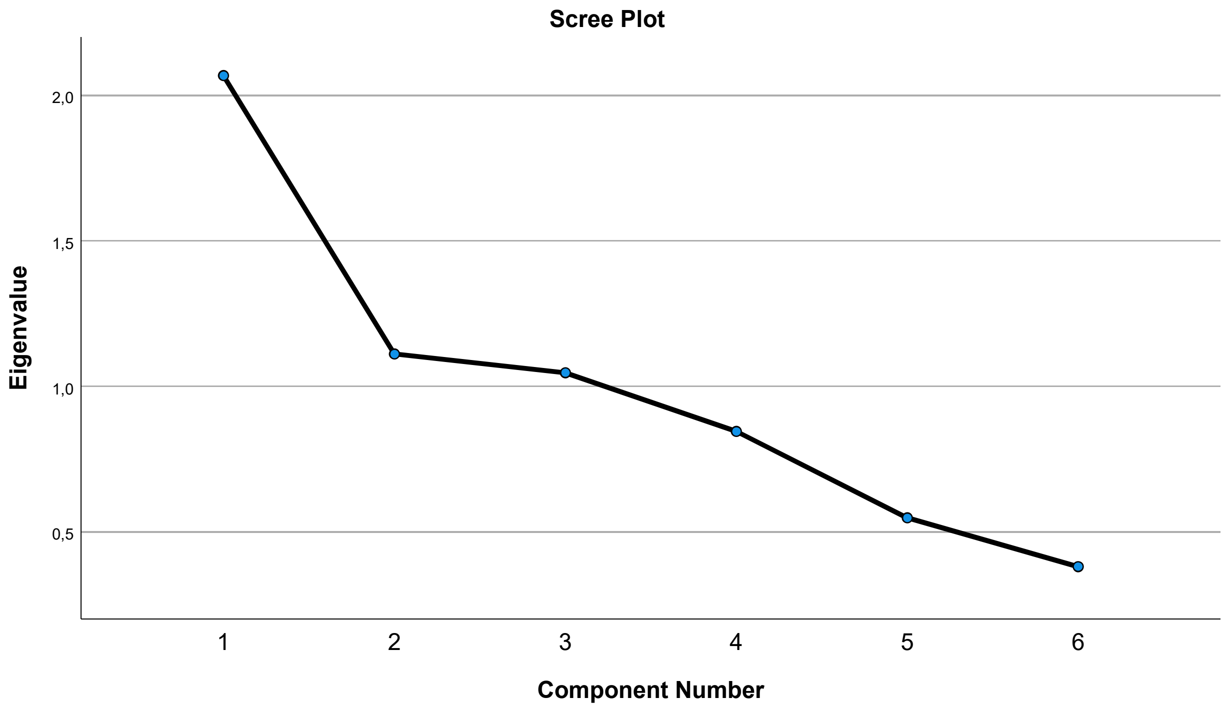

Component Matrix[a]

	Component		
	1	2	3
7	,433	,510	,148
8	-,019	,520	,757
9	,033	-,707	,586
10	,849	,079	-,105
11	,755	-,263	,235
12	,767	-,073	-,204

Extraction Method: Principal Component Analysis.

a. 3 components extracted.

Rotated Component Matrix[a]

	Component		
	1	2	3
7	,383	,493	-,283
8	-,130	,905	,095
9	,016	-,010	,919
10	,849	,073	-,112
11	,741	,097	,367
12	,787	-,110	-,061

Extraction Method: Principal Component Analysis.
Rotation Method: Varimax with Kaiser Normalization.[a]

a. Rotation converged in 5 iterations.

Component Transformation Matrix

Component	1	2	3
1	,993	,119	,020
2	-,062	,646	-,761
3	-,103	,754	,648

Extraction Method: Principal Component Analysis.
Rotation Method: Varimax with Kaiser Normalization.
