# Supplementary material for: Managing disrupted supply chains in Swedish hospitals during the COVID-19 pandemic
Source: Health Syst (Basingstoke). 2024 May 7;14(1):58–68. doi: 10.1080/20476965.2024.2349816 (PMC11843631; doi:10.1080/20476965.2024.2349816)
Supplement: Supplemental Material [file THSS_A_2349816_SM1633.zip › PCA_other units during first wave.pdf]

Factor Analysis

KMO and Bartlett's Test

Kaiser-Meyer-Olkin Measure of Sampling Adequacy.		,704
Bartlett's Test of Sphericity	Approx. Chi-Square	217,060
	df	15
	Sig.	,000

Communalities

	Initial	Extraction
1	1,000	,734
2	1,000	,290
3	1,000	,768
4	1,000	,740
5	1,000	,667
6	1,000	,673

Extraction Method: Principal Component Analysis.

Total Variance Explained

Component	Initial Eigenvalues			Extraction Sums of Squared Loadings		
	Total	% of Variance	Cumulative %	Total	% of Variance	Cumulative %
1	2,598	43,296	43,296	2,598	43,296	43,296
2	1,274	21,232	64,528	1,274	21,232	64,528
3	,875	14,590	79,118			
4	,541	9,015	88,133			
5	,397	6,621	94,754			
6	,315	5,246	100,000			

Total Variance Explained

Component	Rotation Sums of Squared Loadings		
	Total	% of Variance	Cumulative %
1	2,106	35,100	35,100
2	1,766	29,428	64,528
3			
4			
5			
6			

Extraction Method: Principal Component Analysis.

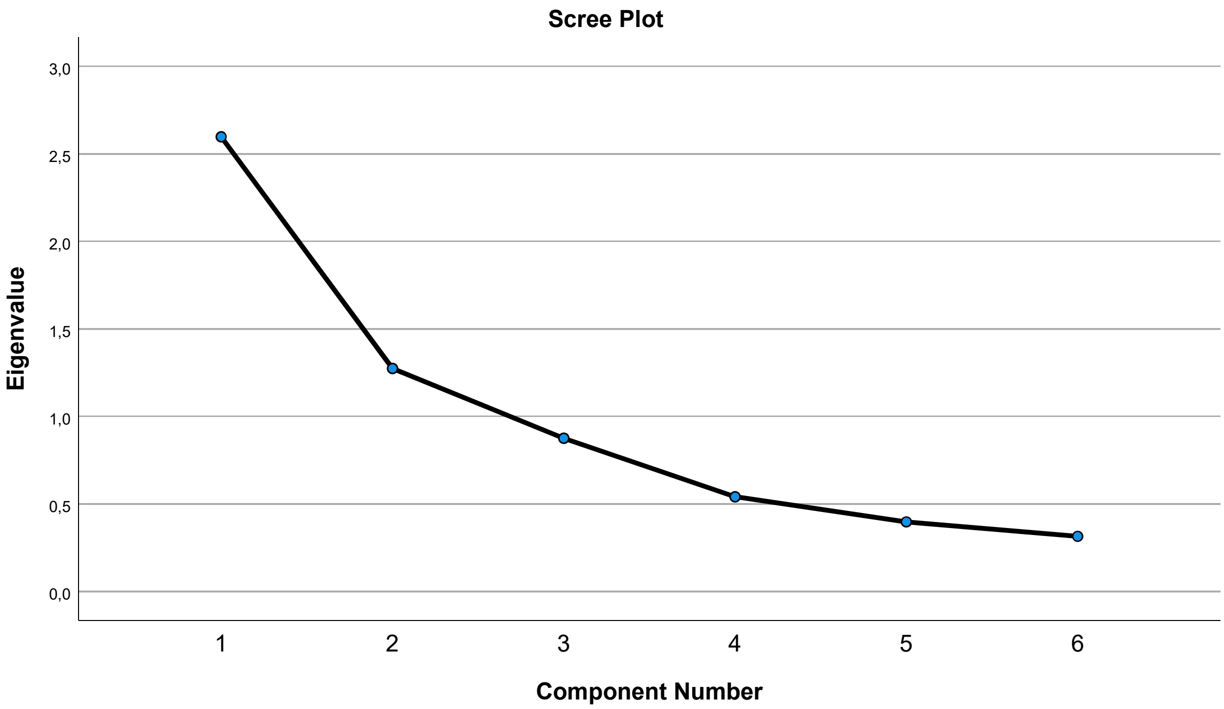

Component Matrix[a]

	Component	
	1	2
1	,753	,408
2	,351	-,408
3	,290	,827
4	,855	,091
5	,786	-,222
6	,689	-,446

Extraction Method: Principal Component Analysis.

a. 2 components extracted.

Rotated Component Matrix[a]

	Component	
	1	2
1	,349	,782
2	,527	-,110
3	-,275	,832
4	,622	,594
5	,758	,303
6	,818	,066

Extraction Method: Principal Component Analysis.
Rotation Method: Varimax with Kaiser Normalization.[a]

a. Rotation converged in 3 iterations.

Component Transformation Matrix

Component	1	2
1	,793	,609
2	-,609	,793

Extraction Method: Principal Component Analysis.
Rotation Method: Varimax with Kaiser Normalization.
