# Supplementary material for: Managing disrupted supply chains in Swedish hospitals during the COVID-19 pandemic
Source: Health Syst (Basingstoke). 2024 May 7;14(1):58–68. doi: 10.1080/20476965.2024.2349816 (PMC11843631; doi:10.1080/20476965.2024.2349816)
Supplement: Supplemental Material [file THSS_A_2349816_SM1633.zip › PCA_other units during later waves.pdf]

Factor Analysis

KMO and Bartlett's Test

Kaiser-Meyer-Olkin Measure of Sampling Adequacy.		,745
Bartlett's Test of Sphericity	Approx. Chi-Square	176,018
	df	15
	Sig.	,000

Communalities

	Initial	Extraction
7	1,000	,605
8	1,000	,158
9	1,000	,099
10	1,000	,579
11	1,000	,583
12	1,000	,580

Extraction Method: Principal Component Analysis.

Total Variance Explained

	Initial Eigenvalues			Extraction Sums of Squared Loadings		
Component	Total	% of Variance	Cumulative %	Total	% of Variance	Cumulative %
1	2,604	43,401	43,401	2,604	43,401	43,401
2	,950	15,830	59,230			
3	,921	15,347	74,578			
4	,705	11,752	86,330			
5	,456	7,606	93,936			
6	,364	6,064	100,000			

Extraction Method: Principal Component Analysis.

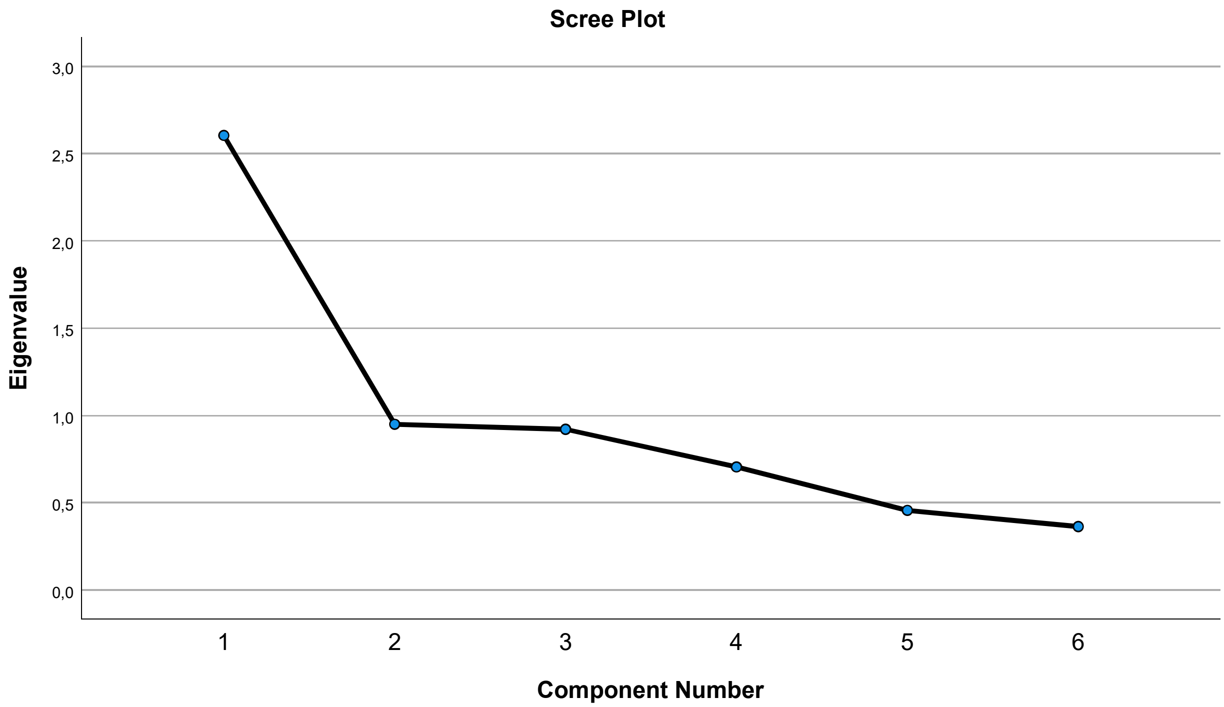

Component Matrix[a]

	Component
	1
7	,778
8	,397
9	,315
10	,761
11	,763
12	,762

Extraction Method: Principal Component Analysis.

a. 1 components extracted.

Rotated Component Matrix[a]

a. Only one component was extracted. The solution cannot be rotated.
