# Supplementary material for: Managing disrupted supply chains in Swedish hospitals during the COVID-19 pandemic
Source: Health Syst (Basingstoke). 2024 May 7;14(1):58–68. doi: 10.1080/20476965.2024.2349816 (PMC11843631; doi:10.1080/20476965.2024.2349816)
Supplement: Supplemental Material [file THSS_A_2349816_SM1633.zip › Questionnaire evasys_in swedish.docx]

|  |  |  |
| --- | --- | --- |
|  | | |

Markera på följande Godkänd ändring:

Använd helst kulspets eller tunn filtpenna vid markering. Formuläret kommer att behandlas automatiskt i systemet. Följ exemplen på vänstra sidan för att optimera inläsningsresultatet.

1. Bakgrundsfrågor

1.1 I vilken typ av verksamhet var du chef under Covid-19 pandemin?

Intensivvård och/eller intermediär vård

Slutenvård geriatrik Öppenvård

Slutenvård medicinsk specialitet

Nyöppnad avdelning för patienter med Covid-19

Annan typ av verksamhet

Slutenvård opererande specialitet Akutmottagning

Jag var inte anställd som chef under Covid-19 pandemin

| 1.2 | Vilka personalkategorier var du chef | | över under Covid-19 pandemin. (Fl | era val är möjliga) |
| --- | --- | --- | --- | --- |
|  |  | Chefer  Arbetsterapeuter Undersköterskor  Icke vårdutbildad personal | Läkare  Fysioterapeuter Vårdbiträde | Sjuksköterskor  Kuratorer Administrativ personal |

1.3 Under vilken period vårdades patienter med Covid-19 på din enhet?

Under hela Covid-19

pandemin (hittills)

Vi behandlade inte patienter med Covid-19

Enbart under första vågen

Enbart under andra och/eller följande vågor

1.4 Vilken region arbetade du i ? Region Skåne

Region Uppsala Region Jönköpings län Region Gotland Region Värmland Region Dalarna

Region Jämtland Härjedalen

Västra Götalandsregionen Region Sörmland

Region Kronoberg Region Blekinge Region Örebro län Region Gävleborg Region Västerbotten

Region Stockholm Region Östergötland Region Kalmar län Region Halland Region Västmanland Region Västernorrland Region Norrbotten

1.5 Inom vilken sjukhusform arbetade du under Covid-19 pandemin? Akutsjukhus Länsdelssjukhus

Regionsjukhus Universitetssjukhus

Länssjukhus Annat

1.6 Du får gärna kommentera bakgrundsfrågorna här;

2. Vi hade kunskap om sjukdomsförloppet och/eller vårdbehovet hos patienter med Covid-19;

2.1 Under Covid-19 pandemins första våg Instämmer

inte alls

Instämmer helt

Ej tillämpbart/ vet ej

2.2 Under Covid-19 pandemins andra våg och senare

Instämmer inte alls

Instämmer helt

Ej tillämpbart/ vet ej

3. Arbetsuppgifterna som vår enhet skulle utföra under Covid-19 pandemin var tydlig;

3.1 Under Covid-19 pandemins första våg Instämmer

inte alls

Instämmer helt

Ej tillämpbart/ vet ej

3.2 Under Covid-19 pandemins andra våg och senare

Instämmer inte alls

Instämmer helt

Ej tillämpbart/ vet ej

4. Kris och beredskapsplanen gjorde att vi kände oss väl förberedda på vår enhet;


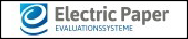


Enkät angående hur sjukvården gjorde för att få balans mellan behov och kapacitet under

EvaSys

4. Kris och beredskapsplanen gjorde att vi kände oss väl förberedda på vår enhet; [Fortsätt]

4.1 Under Covid-19 pandemins första våg Instämmer

inte alls

Instämmer helt

Ej tillämpbart/ vet ej

4.2 Under Covid-19 pandemins andra våg och senare

Instämmer inte alls

Instämmer helt

Ej tillämpbart/ vet ej

5. Du får gärna kommentera de första frågorna här;

5.1

6. Vi hade tillräckligt med personal (sjuksköterskor, undersköterskor, arbetsterapeuter, fysioterapeuter och vårdbiträde) till vården av patienter med Covid-19;

6.1 Under Covid-19 pandemins första våg Instämmer

inte alls

Instämmer helt

Ej tillämpbart/ vet ej

6.2 Under Covid-19 pandemins andra våg och senare

Instämmer inte alls

Instämmer helt

Ej tillämpbart/ vet ej

7. Vårdpersonalen (sjuksköterskor, undersköterskor, arbetsterapeuter, fysioterapeuter, vårdbiträde) hade relevant kompetens för vården av patienter med Covid-19;

7.1 Under Covid-19 pandemins första våg Instämmer

inte alls

Instämmer helt

Ej tillämpbart/ vet ej

7.2 Under Covid-19 pandemins andra våg och senare

Instämmer inte alls

Instämmer helt

Ej tillämpbart/ vet ej

8. Vi hade tillräckligt med läkare till vården av patienter med Covid-19;

8.1 Under Covid-19 pandemins första våg Instämmer

inte alls

Instämmer helt

Ej tillämpbart/ vet ej

8.2 Under Covid-19 pandemins andra våg och senare

Instämmer inte alls

Instämmer helt

Ej tillämpbart/ vet ej

9. Läkarna hade relevant kompetens för vården av patienter med Covid-19;

9.1 Under Covid-19 pandemins första våg Instämmer

inte alls

Instämmer helt

Ej tillämpbart/ vet ej

9.2 Under Covid-19 pandemins andra våg och senare

Instämmer inte alls

Instämmer helt

Ej tillämpbart/ vet ej

10. Vi hade personal som var beordrade från någon annan enhet;

10.1 Under Covid-19 pandemins första våg Inte alls

I mycket hög grad

Ej tillämpbart/ vet ej

10.2 Under Covid-19 pandemins andra våg och senare

Inte alls

I mycket hög grad

Ej tillämpbart/ vet ej

11. Vi hade personal från andra enheter som tjänstgjorde frivilligt på vår enhet;

11.1 Under Covid-19 pandemins första våg Inte alls

I mycket hög grad

Ej tillämpbart/ vet ej

11.2 Under Covid-19 pandemins andra våg och senare

Inte alls

I mycket hög grad

Ej tillämpbart/ vet ej

12. Vi nyanställde medicin-/vårdutbildad personal;

- 1. Under Covid-19 pandemins första våg Inte alls

I mycket hög grad

Ej tillämpbart/ vet ej

- 1. Under Covid-19 pandemins andra våg och senare

Inte alls

I mycket hög grad

Ej tillämpbart/ vet ej


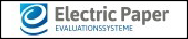


Enkät angående hur sjukvården gjorde för att få balans mellan behov och kapacitet under

EvaSys

13. Vi nyanställde personal som inte var medicin- /vårdutbildad

13.1 Under Covid-19 pandemins första våg Inte alls

I mycket hög grad

Ej tillämpbart/ vet ej

13.2 Under Covid-19 pandemins andra våg och senare

Inte alls

I mycket hög grad

Ej tillämpbart/ vet ej

14. Vi hyrde in personal från bemanningsföretag;

14.1 Under Covid-19 pandemins första våg Inte alls

I mycket hög grad

Ej tillämpbart/ vet ej

14.2 Under Covid-19 pandemins andra våg och senare

Inte alls

I mycket hög grad

Ej tillämpbart/ vet ej

15. Vi anlitade personal från någon typ av intern poolverksamhet;

15.1 Under Covid-19 pandemins första våg Inte alls

I mycket hög grad

Ej tillämpbart/ vet ej

15.2 Under Covid-19 pandemins andra våg och senare

Inte alls

I mycket hög grad

Ej tillämpbart/ vet ej

16. Personal från privata vårdgivare tjänstgjorde hos oss;

16.1 Under Covid-19 pandemins första våg Inte alls

I mycket hög grad

Ej tillämpbart/ vet ej

16.2 Under Covid-19 pandemins andra våg och senare

Inte alls

I mycket hög grad

Ej tillämpbart/ vet ej

17. Vi anlitade volontärer;

17.1 Under Covid-19 pandemins första våg Inte alls

I mycket hög grad

Ej tillämpbart/ vet ej

17.2 Under Covid-19 pandemins andra våg och senare

Inte alls

I mycket hög grad

Ej tillämpbart/ vet ej

18. Du får gärna kommentera personalförsörjningen;

18.1

19. Vi arbetade mer övertid än normalt;

19.1 Under Covid-19 pandemins första våg Instämmer

inte alls

Instämmer helt

Ej tillämpbart/ vet ej

19.2 Under Covid-19 pandemins andra våg och senare

Instämmer inte alls

Instämmer helt

Ej tillämpbart/ vet ej

20. Vi ställde in utbildning och konferenser som inte var Covid-19 relaterat;

20.1 Under Covid-19 pandemins första våg Instämmer

inte alls

Instämmer helt

Ej tillämpbart/ vet ej

20.2 Under Covid-19 pandemins andra våg och senare

Instämmer inte alls

Instämmer helt

Ej tillämpbart/ vet ej

21. Vi satte in extra vårdlag och/eller jourlinjer;

21.1 Under Covid-19 pandemins första våg Instämmer

inte alls

Instämmer helt

Ej tillämpbart/ vet ej

21.2 Under Covid-19 pandemins andra våg och senare

Instämmer inte alls

Instämmer helt

Ej tillämpbart/ vet ej

22. Vi förlängde arbetsskiften;


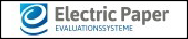


Enkät angående hur sjukvården gjorde för att få balans mellan behov och kapacitet under

EvaSys

| 22. Vi förlängde arbetsskiften; [Fortsätt] |  | | |
| --- | --- | --- | --- |
| 22.1 Under Covid-19 pandemins första våg | Instämmer inte alls | Instämmer helt | Ej tillämpbart/ vet ej |
| 22.2 Under Covid-19 pandemins andra våg | Instämmer | Instämmer | Ej tillämpbart/ |
| och senare | inte alls | helt | vet ej |
|  |  |  |  |
| 23. Vi förkortade arbetsskiften; |  |  |  |
| 23.1 Under Covid-19 pandemins första våg | Instämmer inte alls | Instämmer helt | Ej tillämpbart/ vet ej |
| 23.2 Under Covid-19 pandemins andra våg | instämmer | instämmer | Ej tillämpbart/ |
| och senare | inte alls | helt | vet ej |

24. Du får gärna kommentera om hur ni förändrade användningen av tid under pandemin;

24.1

25. Personalens egna insjuknande i Covid-19 påverkade verksamheten;

25.1 Under Covid-19 pandemins första våg Inte alls

I mycket hög grad

Ej tillämpbart/ vet ej

25.3 Om de blev smittade hur blev de i huvudsak smittade?

25.2 Under Covid-19 pandemins andra våg och senare

Inte alls

I mycket hög grad

Ej tillämpbart/ vet ej

Av patienter

Ej tillämpbart/vet ej

Av kollegor

Hemma eller i samhället

26. Personalen var rädda för att bli smittade eller för att smitta sin familj;

26.1 Under Covid-19 pandemins första våg Inte alls

I mycket hög grad

Ej tillämpbart/ vet ej

26.2 Under Covid-19 pandemins andra våg och senare

Inte alls

I mycket hög grad

Ej tillämpbart/ vet ej

27. Personal var sjukskriven på grund av utmattning eller annan psykisk ohälsa;

27.1 Under Covid-19 pandemins första våg Inte alls

I mycket hög grad

Ej tillämpbart/ vet ej

27.2 Under Covid-19 pandemins andra våg och senare

Inte alls

I mycket hög grad

Ej tillämpbart/ vet ej

28. Personal var sjukskriven på grund av fysisk ohälsa annan än Covid-19 infektion;

28.1 Under Covid-19 pandemins första våg Inte alls

I mycket hög grad

Ej tillämpbart/ vet ej

28.2 Under Covid-19 pandemins andra våg och senare

Inte alls

I mycket hög grad

Ej tillämpbart/ vet ej

29. Personal som tillhörde en riskgrupp omflyttades och arbetade inte med patienter som hade Covid-19;

29.1 Under Covid-19 pandemins första våg Inte alls

I mycket hög grad

Ej tillämpbart/ vet ej

29.2 Under Covid-19 pandemins andra våg och senare

inte alls

i mycket hög grad

Ej tillämpbart/ vet ej

30. Vi satt tätare i personalutrymmen som fikarum, omklädningsrum och konferensrum än vad folkhälsomyndigheterna rekommenderade;


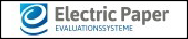


Enkät angående hur sjukvården gjorde för att få balans mellan behov och kapacitet under

EvaSys

30. Vi satt tätare i personalutrymmen som fikarum, omklädningsrum och konferensrum än vad folkhälsomyndigheterna rekommenderade; [Fortsätt]

30.1 Under Covid-19 pandemins första våg Inte alls

I mycket hög grad

Ej tillämpbart/ vet ej

30.2 Under Covid-19 pandemins andra våg och senare

Inte alls

I mycket hög grad

Ej tillämpbart/ vet ej

31. Personalen hade möjlighet att få stöd av kuratorer, psykologer och/eller präster;

31.1 Under Covid-19 pandemins första våg Inte alls

I mycket hög grad

Ej tillämpbart/ vet ej

31.2 Under Covid-19 pandemins andra våg och senare

Inte alls

I mycket hög grad

Ej tillämpbart/ vet ej

32. Du får gärna kommentera om personal;

32.1

33. Vi hade brist på skyddsutrustning;

33.1 Under Covid-19 pandemins första våg Inte alls

I mycket hög grad

Ej tillämpbart/ vet ej

33.2 Under Covid-19 pandemins andra våg och senare

Inte alls

I mycket hög grad

Ej tillämpbart/ vet ej

34. Vi använde säkrare skyddsutrustning än vad som rekommenderades;

34.1 Under Covid-19 pandemins första våg Inte alls

I mycket hög grad

Ej tillämpbart/ vet ej

34.2 Under Covid-19 pandemins andra våg och senare

Inte alls

I mycket hög grad

Ej tillämpbart/ vet ej

35. Vi använde mindre säker utrustning än vad som rekommenderades;

35.1 Under Covid-19 pandemins första våg Inte alls

I mycket hög grad

Ej tillämpbart/ vet ej

35.2 Under Covid-19 pandemins andra våg och senare

Inte alls

I mycket hög grad

Ej tillämpbart/ vet ej

36. Du får gärna kommentera angående skyddsutrustning här;

36.1

37. Vi hade brist på förbrukningsartiklar såsom provtagningspinnar, slangar till respiratorer mm.;

37.1 Under Covid-19 pandemins första våg Inte alls

I mycket hög grad

Ej tillämpbart/ vet ej

37.2 Under Covid-19 pandemins andra våg och senare

Inte alls

I mycket hög grad

Ej tillämpbart/ vet ej

38. Vi hade brist på medicinteknisk utrustning;

38.1 Under Covid-19 pandemins första våg Inte alls

I mycket hög grad

Ej tillämpbart/ vet ej


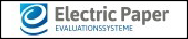


Enkät angående hur sjukvården gjorde för att få balans mellan behov och kapacitet under

EvaSys

Ej tillämpbart/ vet ej

I mycket hög grad

Inte alls

38.2 Under Covid-19 pandemins andra våg och senare

38. Vi hade brist på medicinteknisk utrustning; [Fortsätt]

39. Vi hade brist på läkemedel till behandling av Covid-19 patienterna;

39.1 Under Covid-19 pandemins första våg Inte alls

I mycket hög grad

Ej tillämpbart/ vet ej

39.2 Under Covid-19 pandemins andra våg och senare

Inte alls

I mycket hög grad

Ej tillämpbart/ vet ej

40. Vi hade brist på utrymme;

40.1 Under Covid-19 pandemins första våg Inte alls

I mycket hög grad

Ej tillämpbart/ vet ej

40.2 Under Covid-19 pandemins andra våg och senare

Inte alls

I mycket hög grad

Ej tillämpbart/ vet ej

41. Vi byggde om i lokalerna på vår enhet för att kunna hantera patienter med Covid-19;

41.1 Under Covid-19 pandemins första våg Inte alls

I mycket hög grad

Ej tillämpbart/ vet ej

41.2 Under Covid-19 pandemins andra våg och senare

Inte alls

I mycket hög grad

Ej tillämpbart/ vet ej

42. Du får gärna kommentera bristerna här;

42.1

43. Jag hade digitala möten med mina chefer;

43.1 Under Covid-19 pandemins första våg Inte alls

I mycket hög grad

Ej tillämpbart/ vet ej

43.2 Under Covid-19 pandemins andra våg och senare

Inte alls

I mycket hög grad

Ej tillämpbart/ vet ej

44. Jag hade digitala möten med min personal;

44.1 Under Covid-19 pandemins första våg Inte alls

I mycket hög grad

Ej tillämpbart/ vet ej

44.2 Under Covid-19 pandemins andra våg och senare

Inte alls

I mycket hög grad

Ej tillämpbart/ vet ej

45. Jag hade dagliga möten med personalen på min enhet;

45.1 Under Covid-19 pandemins första våg Inte alls

I mycket hög grad

Ej tillämpbart/ vet ej

45.2 Under Covid-19 pandemins andra våg och senare

Inte alls

I mycket hög grad

Ej tillämpbart/ vet ej

46. Min chef, jag och mina chefskollegor hade dagliga möten;

46.1 Under Covid-19 pandemins första våg Inte alls

I mycket hög grad

Ej tillämpbart/ vet ej

46.2 Under Covid-19 pandemins andra våg och senare

Inte alls

I mycket hög grad

Ej tillämpbart/ vet ej

47. Jag fick tillräckligt med information från min chef;

47.1 Under Covid-19 pandemins första våg Instämmer

Instämmer

Ej tillämpbart/


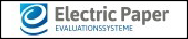


Enkät angående hur sjukvården gjorde för att få balans mellan behov och kapacitet under

EvaSys

Ej tillämpbart/ vet ej

Instämmer helt

Instämmer inte alls

47.2 Under Covid-19 pandemins andra våg och senare

47. Jag fick tillräckligt med information från min chef; [Fortsätt]

48. Jag fick tillräckligt med information från sjukhusledningen;

48.1 Under Covid-19 pandemins första våg Instämmer

inte alls

Instämmer helt

Ej tillämpbart/ vet ej

48.2 Under Covid-19 pandemins andra våg och senare

Instämmer inte alls

Instämmer helt

Ej tillämpbart/ vet ej

49. Min chef var mer närvarande än vad hen brukar vara på min enhet;

49.1 Under Covid-19 pandemins första våg Instämmer

inte alls

Instämmer helt

Ej tillämpbart/ vet ej

49.2 Under Covid-19 pandemins andra våg och senare

Instämmer inte alls

Instämmer helt

Ej tillämpbart/ vet ej

50. Jag var mer närvarande än vad jag brukar vara på min enhet;

50.1 Under Covid-19 pandemins första våg Instämmer

inte alls

Instämmer helt

Ej tillämpbart/ vet ej

50.2 Under Covid-19 pandemins andra våg och senare

Instämmer inte alls

Instämmer helt

Ej tillämpbart/ vet ej

51. Du får gärna kommentera här;

51.1

52. Vi visste hur vi skulle använda den personliga skyddsutrustningen;

52.1 Under Covid-19 pandemins första våg Instämmer

inte alls

Instämmer helt

Ej tillämpbart/ vet ej

52.2 Under Covid-19 pandemins andra våg och senare

Instämmer inte alls

Instämmer helt

Ej tillämpbart/ vet ej

53. Pandemiövningar har bidragit till att vi var väl förberedda;

53.1 Under Covid-19 pandemins första våg Instämmer

inte alls

Instämmer helt

Ej tillämpbart/ vet ej

53.2 Under Covid-19 pandemins andra våg och senare

Instämmer inte alls

Instämmer helt

Ej tillämpbart/ vet ej

54. Utvärderingar av pandemier och/eller nuvarande pandemi har bidragit till att vi var väl förberedda;

54.1 Under Covid-19 pandemins första våg Instämmer

inte alls

Instämmer heltt

Ej tillämpbart/ vet ej

54.2 Under Covid-19 pandemins andra våg och senare

Instämmer inte alls

Instämmer helt

Ej tillämpbart/ vet ej

55. Vi har varit på konferenser och/eller utbildningar som bidragit till att vi var väl förberedda;

55.1 Under Covid-19 pandemins första våg Instämmer

inte alls

Instämmer helt

Ej tillämpbart/ vet ej

55.2 Under Covid-19 pandemins andra våg och senare

Instämmer inte alls

Instämmer hetl

Ej tillämpbart/ vet ej

56. Vi fick tillräcklig utbildning om Covid-19;

56.1 Under Covid-19 pandemins första våg Instämmer

Instämmer

Ej tillämpbart/

Ej tillämpbart/ vet ej

Instämmer helt

Instämmer inte alls

56.2 Under Covid-19 pandemins andra våg och senare

56. Vi fick tillräcklig utbildning om Covid-19; [Fortsätt]

57. Du får gärna kommentera om förberedelse här;

57.1

58. Vi fick den service av laboratorier som behövdes för behandling och vård;

58.1 Under Covid-19 pandemins första våg Instämmer

inte alls

Instämmer helt

Ej tillämpbart/ vet ej

58.2 Under Covid-19 pandemins andra våg och senare

Instämmer inte alls

Instämmer helt

Ej tillämpbart/ vet ej

59. Vi fick den service av andra enheter som tex inköp, förråd, sterilcentral som vi behövde;

59.1 Under Covid-19 pandemins första våg Instämmer

inte alls

Instämmer helt

Ej tillämpbart/ vet ej

59.2 Under Covid-19 pandemins andra våg och senare

Instämmer inte alls

Instämmer helt

Ej tillämpbart/ vet ej

60. Vi fick det stöd av sjukhusledningen som vi behövde;

60.1 Under Covid-19 pandemins första våg Instämmer

inte alls

Instämmer helt

Ej tillämpbart/ vet ej

60.2 Under Covid-19 pandemins andra våg och senare

Instämmer inte alls

Instämmer helt

Ej tillämpbart/ vet ej

61. Vi använde Folkhälsomyndighetens prognoser till våra beslut om vilken vårdkapacitet vi skulle bygga upp till Covid-19 patienterna;

61.1 Under Covid-19 pandemins första våg Inte alls

I mycket hög grad

Ej tillämpbart/ vet ej

61.2 Under Covid-19 pandemins andra våg och senare

Inte alls

I mycket hög grad

Ej tillämpbart/ vet ej

62. Vi använde regionala prognoser till våra beslut om vilken vårdkapacitet vi skulle bygga upp till Covid-19 patienterna;

62.1 Under Covid-19 pandemins första våg Inte alls

I mycket hög grad

Ej tillämpbart/ vet ej

62.2 Under Covid-19 pandemins andra våg och senare

Inte alls

I mycket hög grad

Ej tillämpbart/ vet ej

63. Vi använde lokala prognoser till våra beslut om vilken vårdkapacitet vi skulle bygga upp till Covid-19 patienterna;

63.1 Under Covid-19 pandemins första våg Inte alls

I mycket hög grad

Ej tillämpbart/ vet ej

63.2 Under Covid-19 pandemins andra våg och senare

Inte alls

I mycket hög grad

Ej tillämpbart/ vet ej

64. Vi använde en procentuell andel av behovet i tidigare vågor till våra beslut om vilken vårdkapacitet vi skulle bygga upp till Covid-19 patienterna i våg två eller senare;

64.1 Under Covid-19 pandemins andra våg och senare

Inte alls

I mycket hög grad

Ej tillämpbart/ vet ej

65. Vi använde medelvårdtid för patienterna i vår verksamhet till våra beslut om vilken vårdkapacitet vi skulle bygga upp till Covid-19 patienterna;

65.1 Under Covid-19 pandemins första våg Inte alls

I mycket hög grad

Ej tillämpbart/ vet ej

65.2 Under Covid-19 pandemins andra våg eller senare

Inte alls

I mycket hög grad

Ej tillämpbart/ vet ej

66. Vi använde den förväntade sjukfrånvaron hos vår egen personal under Covid-19 pandemin till våra beslut om vilken vårdkapacitet vi skulle bygga upp till Covid-19 patienterna;

66.1 Under Covid-19 pandemins första våg Inte alls

I mycket hög grad

Ej tillämpbart/ vet ej

66.2 Under Covid-19 pandemins andra våg eller senare

Inte alls

I mycket hög grad

Ej tillämpbart/ vet ej

67. Du får gärna kommentera om underlagen för er kapacitetsuppbyggnad;

67.1

68. Jag är nöjd med hur sjukhuset löste materialförsörjningen;

68.1 Under Covid-19 pandemins första våg Instämmer

inte alls

Instämmer helt

Ej tillämpbart/ vet ej

68.2 Under Covid-19 pandemins andra våg och senare

Instämmer inte alls

Instämmer helt

Ej tillämpbart/ vet ej

69. Jag är nöjd med hur sjukhuset löste personalförsörjningen;

69.1 Under Covid-19 pandemins första våg Instämmer

inte alls

Instämmer helt

Ej tillämpbart/ vet ej

69.2 Under Covid-19 pandemins andra våg och senare

Instämmer inte alls

Instämmer helt

Ej tillämpbart/ vet ej

70. Jag är nöjd med sjukhusets krisorganisation;

70.1 Under Covid-19 pandemins första våg Instämmer

inte alls

Instämmer helt

Ej tillämpbart/ vet ej

70.2 Under pandemins andra våg och senare Instämmer

inte alls

Instämmer helt

Ej tillämpbart/ vet ej

71. Jag är nöjd med hur sjukhuset skötte intern och/eller extern kommunikation;

71.1 Under Covid-19 pandemins första våg Instämmer

inte alls

Instämmer helt

Ej tillämpbart/ vet ej

71.2 Under Covid-19 pandemins andra våg och senare

Instämmer inte alls

Instämmer helt

Ej tillämpbart/ vet ej

72. Min enhet var väl förberedd för Covid-19 pandemin;

72.1 Under Covid-19 pandemins första våg Instämmer

inte alls

Instämmer helt

Ej tillämpbart/ vet ej

72.2 Under Covid-19 pandemins andra våg och senare

Instämmer inte alls

Instämmer helt

Ej tillämpbart/ vet ej

73. Min enhet klarade av Covid-19 pandemin på ett bra sätt;

- 1. Under Covid-19 pandemins första våg Instämmer

inte alls

Instämmer helt

Ej tillämpbart/ vet ej

- 1. Under Covid-19 pandemins andra våg och senare

Instämmer inte alls

Instämmer helt

Ej tillämpbart/ vet ej

# 74. Du får gärna kommentera mer här;

74.1
